# Enhancing Blue Emission in Poly(*N*‑vinylcarbazole): Synthesis, Functionalization with Anthracene, and Mitigation of Aggregation-Caused Quenching

**DOI:** 10.1021/acsomega.5c06357

**Published:** 2025-08-21

**Authors:** Daniela Corrêa Santos, Gabriel de Sousa Barros, Maria de Fátima Vieira Marques

**Affiliations:** Instituto de Macromoléculas Professora Eloisa Mano, 28125 Universidade Federal do Rio de Janeiro (IMA/UFRJ), Rio de Janeiro, Rio de Janeiro 21941-598, Brazil

## Abstract

This study reports the synthesis and functionalization of poly­(*N*-vinylcarbazole) (PVK) with anthracene units to enhance its blue photoluminescence properties. Structural and thermal analyses confirmed successful incorporation of anthracene moieties into the PVK backbone at an approximate 3:1 ratio of PVK repeat unit to anthracene. Photophysical characterization showed that anthracene-functionalized PVK (PVK–An) retained blue-region emission (432 nm), although with reduced emission efficiency due to π–π stacking interactions. Incorporating PVK–An into an inert poly­(methyl methacrylate) (PMMA) matrix mitigated aggregation-caused quenching (ACQ), increasing the photoluminescence quantum yield (PLQY) from 0.5% to 28.9% in the optimized 70:30 wt % PMMA/PVK–An blend. Time-resolved photoluminescence (TRPL) analysis revealed that the emission in both neat PVK and PVK–An arises from two decay pathways, S_1_ → S_0_ and S_1_* → S_0_, corresponding to singlet excimer states emissions, respectively. Notably, PVK–An exhibited reduced excimer formation, as indicated by shorter fluorescence lifetimes (τ_1_ ≈ 5 ns) and a lower contribution from the longer decay component (τ_2_ ≈ 15 ns, Rel. 54.80%) compared to pristine PVK (τ_2_ ≈ 25 ns, Rel. 74.33%). These findings demonstrate the synergistic benefits of side-chain engineering and morphological control in the design of high-performance blue-emitting polymers for OLED applications.

## Graphical abstract

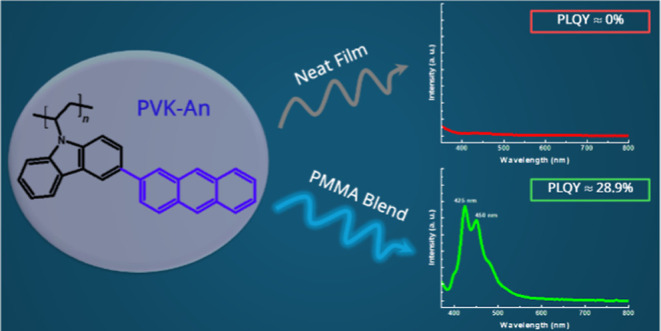

## 1 Introduction

Organic light-emitting diodes (OLEDs) are advanced electroluminescent devices that generate light through the recombination of charge carriers in organic materials under an applied voltage. With their ability to produce vibrant colors, low power consumption, and flexible designs, OLEDs are becoming an increasingly attractive alternative to traditional light-emitting diodes (LEDs) for applications in displays and lighting panels. While LEDs are well-established in the commercial sector, OLEDs offer unique advantages such as higher color purity, lighter weight, and the potential for ultrathin, flexible devices.^1,2^

Despite these advantages, OLED technology still faces significant challenges, particularly in the manufacturing of large-area devices and scaling up production while keeping costs low. These issues stem from the complexity of producing high-quality films with uniform emissive layers using traditional vacuum deposition methods. Solution processing methods such as inkjet printing, roll-to-roll coating, and spin coating have emerged as promising alternatives due to their potential to simplify fabrication, lower costs, and enable the production of flexible and large-area devices. However, these methods demand the development of emissive materials, particularly polymers, capable of forming high-quality films through solution deposition techniques.^3–5^

Among these materials, poly­(*N*-vinylcarbazole) (PVK) stands out as one of the first polymers used in OLED devices. PVK offers several advantages, including straightforward synthesis, short reaction times, and high yields. Its emission in the blue region (410 nm) with a narrow emission band makes it highly desirable for blue-emitting layers in OLEDs.^6^ However, PVK’s low emissive efficiency has been a limiting factor, primarily due to its intrinsic low photoluminescence quantum yield.^7,8^

To overcome this limitation, functionalization strategies involving the incorporation of highly emissive units into the polymer backbone have been proposed. Such modifications aim to enhance the polymer’s photoluminescent properties while preserving its inherent advantages.^9–11^

In 2023, a study explored the functionalization of PVK with fluorene units attached to the pendant groups of the polymer chain. The main goal was to suppress the strong π-electronic coupling between carbazole units, which typically limits the radiative transition efficiency in pure PVK. The functionalization was successfully achieved, resulting in polymers with improved optical properties. These functionalized polymers exhibited deep-blue light emission with shorter wavelengths and enhanced color purity, making them highly suitable for optoelectronic applications. Furthermore, the addition of fluorene improved the stability and performance of the materials in polymer light-emitting devices. Although researchers have demonstrated that functionalizing PVK is an effective strategy for enhancing its emissive behavior, the resulting device exhibited a low EQE, with a maximum of 0.44%. This highlights the importance of exploring alternative functionalization with highly emissive units to achieve OLEDs with improved efficiency.^9^

Therefore, this study focuses on the functionalization of PVK with anthracene to enhance its performance as an emissive material for improved OLED devices. Anthracene is a fused aromatic molecule known for its strong fluorescence in the blue region, making it a promising candidate for enhancing PVK’s emissive efficiency.^12,13^ This work includes the synthesis and characterization of both PVK and its anthracene-functionalized derivative (PVK–An). Characterization techniques such as ^1^H NMR and thermogravimetric analysis (TGA) suggested a 3:1 ratio of carbazole repeat units to anthracene. The incorporation of anthracene induced a strong aggregation-caused quenching (ACQ) effect in solid state, due to the enhanced π–π stacking interactions arising from the extended conjugated system.

However, by mitigating this effect through the introduction of an inert matrix poly­(methyl methacrylate) (PMMA), the matrix containing 30% of PVK–An exhibited improved photophysical behavior, achieving a PLQY of 28.9%, 2.6 times higher than that of pristine PVK. This enhancement was attributed not only to anthracene’s intrinsic emissive capacity but also to the reduced excimer formation, likely resulting from conformational changes induced by functionalization. This interpretation was further supported by time-resolved fluorescence measurements, which showed shorter decay times for PVK–An compared to pristine PVK, consistent with the suppression of excimer-related long fluorescence lifetimes. To date, no published studies have specifically investigated the functionalization of PVK with anthracene as a pendant group on the carbazole units, nor its impact on the charge transport properties and emission performance of PVK. By enhancing PVK’s emissive performance, this research aims to contribute to the development of more efficient and cost-effective materials for next-generation OLED technologies.

## 2 Experimental Section

### 2.1 Materials

Deuterated chloroform, tetrakis­(triphenylphosphine)­palladium(0), boron trifluoride diethyl etherate, *N*-bromosuccinimide, and poly­(methyl methacrylate) (PMMA) were purchased from Sigma-Aldrich Brasil Ltd. Ethanol, acetone, dichloromethane, and toluene were obtained from Vetec Química Fina Ltd., while dimethylformamide (DMF) was acquired from Tedia Brasil Ltd. The reagents 9-vinylcarbazole, 2-anthraceneboronic acid, and Aliquat 366 were supplied by Zhengzhou Alfa Chemical Co., Ltd. (China), and potassium carbonate was obtained from Baker-Analyzed Reagents, J.T.Baker (USA). All solvents were dried prior to use by fractional distillation under a nitrogen atmosphere. Unpatterned indium tin oxide (ITO) glass substrates (15 mm × 20 mm) 14–16 Ω•sq^–1^ were purchased from Ossila Ltd.

Proton nuclear magnetic resonance (^1^H NMR) spectra were recorded on a Bruker Avance III 400 MHz spectrometer at room temperature (25 °C). Samples were dissolved in deuterated chloroform (CDCl_3_), and spectra were acquired. Chemical shifts (δ) are reported in parts per million (ppm) relative to the residual proton signal of CDCl_3_ (δ = 7.26 ppm). UV–vis spectroscopy was performed in a Shimadzu Ltd. model 2600i spectrometer. The photoluminescence studies were recorded using an Edinburgh Instruments Ltd., UK, spectrofluorometer model FS5. The thin films were cleaned using ultrasonic cleaner CS0306 Cleansonic Ltd., and a UV ozone cleaner L2002A3. Films deposition was carried out using a spin coater L2001A3 from Ossila Ltd., UK. Cyclic voltammetry was performed using a PGSTAT302N model FRA32 M Potentiostat from Metrohm Brasil.

### 2.2 Synthesis

#### 2.2.1 Poly­(*N*-vinylcarbazole) (PVK)

PVK was synthesized via cationic polymerization.^14,15^ The reaction was conducted for 10 min in 50 mL of dry dichloromethane as the solvent with of 1 g of 9-vinylcarbazole (5 mmol) and 1.0 mL of boron trifluoride diethyl etherate solution in dichloromethane 0.05 v/v, under an inert atmosphere at 0 °C. Upon completion of the synthesis, the polymer was precipitated in ethanol and isolated by vacuum filtration, affording a white solid with an estimated yield of 80.3% w/w, calculated as the ration between the weight of monomer used and the weight of polymer obtained.

#### 2.2.2 α,ω-Dimethylpoly­[1-(3,6-dibromo-9*H*-carbazol-9-yl)­ethane-1,2-diyl] (PVK–Br)

Bromination was achieved through a nucleophilic substitution (S_N_2) reaction using 0.4 g of the synthesized PVK (1 equiv) and 0.9 g of *N*-bromosuccinimide (NBS) (5 mmol, 2.5 equiv) in 20 mL of dimethylformamide (DMF). The NBS slowly dropped into the reaction flask containing polymer solution keeping a temperature of 0 °C. After the NBS addition, the reaction was performed at room temperature for 24 h under an inert atmosphere. The product was precipitated in ethanol and filtered under vacuum, yielding a light pink solid with an estimated yield of 34.8%, considering the theoretical mass of bromine incorporated into the polymer chain, as determined by the amount of NBS introduced into the reaction medium.^16,17^

#### 2.2.3 PVK Functionalized with Anthracene (PVK–An)

PVK–Br was functionalized via Suzuki–Miyaura cross-coupling reaction. Two mmol of PVK–Br (1 equiv), 4 mmol of 2-anthraceneboronic acid (0.888 g, 2 equiv), 0.02 mmol of palladium-tetrakis­(triphenylphosphine) (Pd­(PPh_3_)_4_) (0.139 g, 0.01 equiv) were added in a flask with 100 mL of dry toluene. Fourteen ml of K_2_CO_3_ aqueous solution 2 mol•dm^–3^ with four drops of Aliquat 366 was sequentially dropped into the bottom flask. The reaction mixture was maintained at 110 °C for 24 h in the dark under an inert atmosphere. The resulting polymer was precipitated in methanol and isolated by vacuum filtration, affording a light-yellow solid with an estimated yield of 90% w/w, calculated from the ratio between the total weight used of the starting materials (without the active ends) and the final product.^9^

### 2.3 Photophysical Investigation

#### 2.3.1 General Procedure

Photoluminescence (PL) measurements were performed using a spectrofluorometer (Edinburgh Instruments, UK) equipped with the SC-10 front-face sample holder module for recording emission spectra, and the SC-30 integrating sphere module for determining the photoluminescence quantum yield (PLQY). The PLQY values were calculated directly using the instrument’s proprietary software (Fluoracle), following standard protocols and including both sample and reference corrections. Time resolved photoluminescence (TRPL) was carried out using a time-correlated single photon counting (TCSPC) with a picosecond pulsed LED with excitation of 340 nm. The investigation was conducted on thin-film samples deposited on quartz substrates, with the preparation procedure described in detail below.

#### 2.3.2 Preparation of Polymer Solutions

Solutions of PVK, PVK–Br, and PVK–An were prepared in tetrahydrofuran (THF) at a concentration of 10 g•l^–1^. These solutions were stirred at room temperature for 12 h to ensure complete dissolution.

#### 2.3.3 Deposition of Neat-Films

The quartz substrates were first cleaned in an ultrasonic bath using acetone and then isopropanol, each for 10 min, followed by ozone treatment for 30 min. Thin films were deposited using spin coating. A volume of 100 μL of each THF solution was deposited on quartz substrates at 2000 rpm for 45 s. Following deposition, an annealing step was performed at 100 °C for 10 min.

#### 2.3.4 Preparation of Polymer Blends

Polymer blend films were prepared by incorporating the synthesized PVK and PVK–An into an optically and electrically inert poly­(methyl methacrylate) (PMMA) matrix at different weight ratios, as detailed in Table 1. PMMA was employed as a neutral host polymer to minimize intermolecular interactions and isolate the intrinsic photophysical behavior of the functionalized materials. The resulting mixtures were deposited onto substrates using the same spin-coating conditions applied to the pure polymers, ensuring uniform film thickness and morphology. This methodology enabled a consistent and reliable comparison of their optoelectronic properties.

**Table 1:** Formulations of Polymer Films Based on PMMA/PVK–An and PMMA/PVK

solution	PMMA/PVK–An (v/v)	solution	PMMA/PVK (v/v)
1	80:20	1	90:10
2	78:22	2	80:20
3	76:24	3	70:30
4	74:26	4	50:50
5	72:28	5	30:70
6	70:30	6	20:80
7	60:40	7	10:90
8	55:45		
9	50:50		

### 2.4 Energy Levels Measurement

#### 2.4.1 Optical Bandgap

The optical bandgap (*E*_g_^opt^) was calculated using the onset wavelength (λ_onset_) of the absorption spectra of the synthesized polymers. The onset was determined by the tangent method using Origin software, based on measurements recorded in thin films. The *E*_g_^opt^ value was obtained using eq 1, where *c* is the speed of light and *h* is the Planck’s constant.^18^

1 $$E_{g}^{opt}=\frac{cxh}{\lambda_{onset}}$$

#### 2.4.2 Energy Levels

The highest occupied molecular orbital (HOMO) and lowest unoccupied molecular orbital (LUMO) energy levels of the synthesized polymers were estimated by combining electrochemical and optical data. The onset oxidation potential (*E*_ox_^onset^) was obtained from cyclic voltammetry (CV) measurements, while the optical bandgap (*E*_g_^opt^) was calculated from the onset of UV–Vis absorption, as described in eq 1. The HOMO energy level was then determined using eq 2, which references the ferrocene/ferrocenium (*F*_c_/*F*_c_^+^) redox couple as an internal standard ( $E_{1/2\left(F_{c}/F_{c}^{+}\right)}$ = 0.0 V vs *F*_c_/*F*_c_^+^) and applies a correction factor of 4.8 eV to align the potential with the vacuum energy level.^19–22^ Once the HOMO energy was determined, the LUMO energy level was calculated by adding the optical bandgap, as shown in eq 3.

2 $$E_{HOMO}=-\left(E_{ox}^{onset}-E_{\frac{1}{2}\left(\frac{F_{c}}{F_{c}^{+}}\right)}+4.8\right)eV$$

3 $$E_{LUMO}=\left(E_{HOMO}+E_{g}^{opt}\right)$$

The polymers were deposited by spin coating onto glass substrates coated with indium tin oxide (ITO), which had been previously cleaned in an ultrasonic bath using acetone and then isopropanol, each for 10 min, followed by ozone treatment for 30 min. Cyclic voltammetry was performed using ITO as the working electrode (WE), silver/silver chloride (Ag/AgCl) as the reference electrode (RE), and a platinum rod as the counter electrode (CE). The measurements were conducted in an electrolyte solution of tetrabutylammonium hexafluorophosphate (TBAPF6) in acetonitrile (0.1 mol·dm^–3^), previously degassed with nitrogen. Ferrocene was also measured as a standard. Analyses were carried out in the range of 2 mV to −2 mV, with a scan rate of 50 mV•s^–1^.

## 3 Results/Discussion

### 3.1 Proton Nuclear Magnetic Resonance (^1^H NMR) Spectroscopy

The NMR analysis revealed the characteristic peaks corresponding to the desired structures (Figure 1). Signals in the downfield region (highlighted in pink, between 6 and 8 ppm) were attributed to the aromatic hydrogens of the polymer backbone. Signals in the high-field region (highlighted in green, 3–0.5 ppm) were attributed to the aliphatic hydrogens of the PVK chain.^15,16,23^

**Figure 1:**
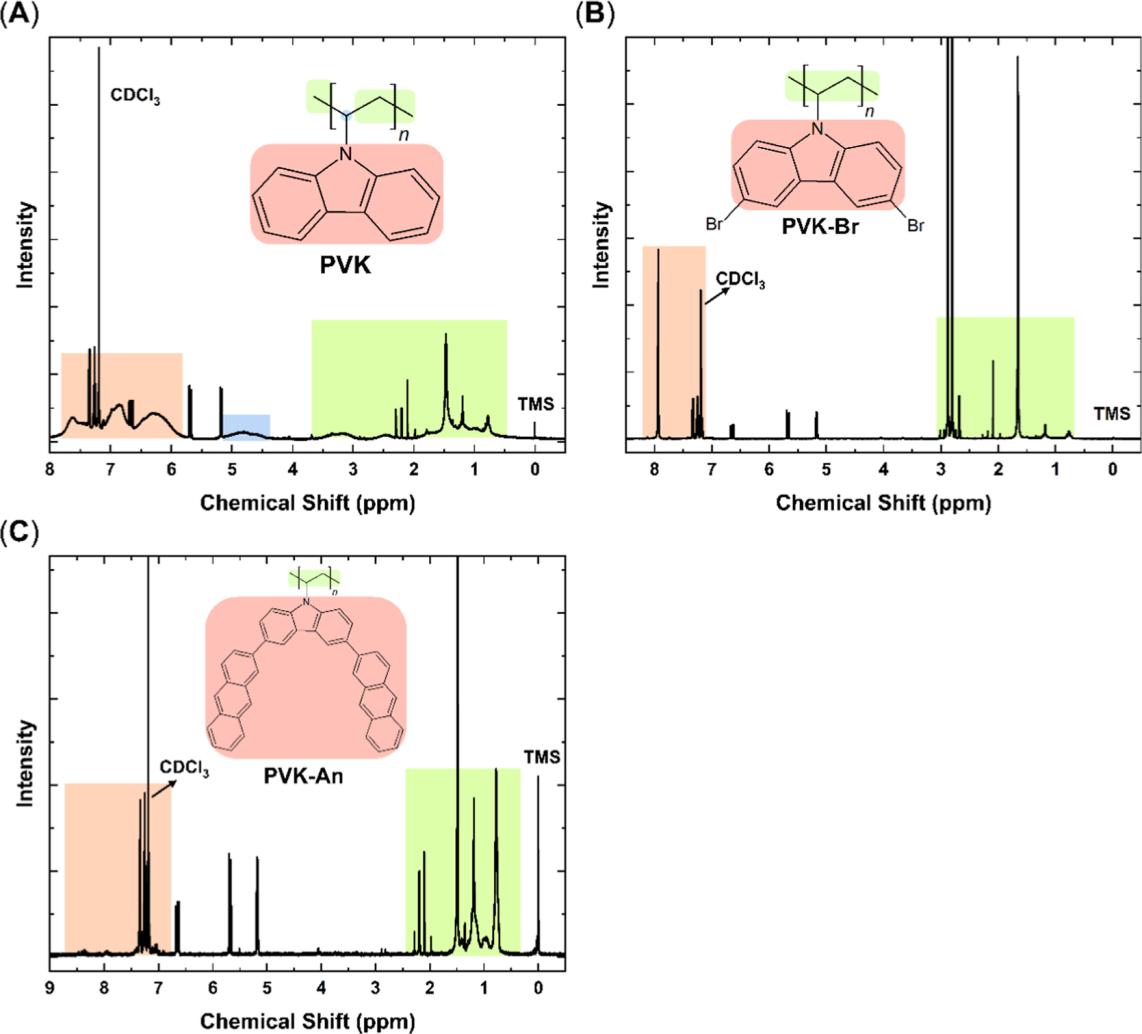
^1^H NMR spectra of (A) PVK, (B) PVK–Br, and (C) PVK–An.

The broadening of the peaks, particularly pronounced in the PVK spectrum, provides strong evidence for polymer chain formation, as such broadening is a typical characteristic of polymeric materials in NMR spectra. This effect can also be attributed to increased chain entanglement, restricted segmental motion, and a broader distribution of magnetic environments associated with dispersity. Similar broadening was observed in the spectra of PVK–Br and PVK–An, although to a lesser extent, likely due to their lower signal intensities compared to PVK.^24^

A prominent peak around 1.5 ppm likely corresponds to residual water, while a peak at approximately 3 ppm, present in the spectrum of PVK–Br, is characteristic of residual DMF solvent. The presence of DMF traces is not unexpected, given its high boiling point, which makes complete removal from the polymer matrix challenging. These findings collectively confirm the successful synthesis and functionalization of the PVK derivatives while also highlighting potential residual impurities from the synthetic process.^24–26^

Figure S1 also provides a detailed view of the region highlighted between 7.8 and 8.6 ppm, showing low-intensity peaks in the aromatic region, characteristic of hydrogen atoms in the anthracene structure.^23^ These peaks confirm the successful functionalization of PVK with anthracene, as such deshielded aromatic protons are absent in the spectrum of pristine PVK, indicating the presence of new chemical environments associated with the anthracene moieties.

Moreover, peaks between 5 and 6 ppm were observed in all samples. These peaks can be attributed to variations in chain-end groups resulting from precipitation in alcoholic solvents (methanol and ethanol). During precipitation, the reaction medium may retain active chain ends within the polymer structure, which can undergo side reactions with the alcoholic solvent, thereby generating different chain-end groups.^27^

### 3.2 Thermogravimetric Analysis (TGA)

The TGA analysis confirmed the high thermal stability of the synthesized PVK, with a decomposition temperature approaching 400 °C, as shown in Figure 2. The TGA curve exhibits a single, well-defined mass-loss event, suggesting efficient polymer synthesis and high chemical purity. The material undergoes nearly complete thermal degradation, with negligible residual mass, which is consistent with the expected degradation pathway of PVK’s molecular structure. The elevated decomposition temperature underscores the intrinsic thermal robustness of PVK, reinforcing its suitability for high-temperature optoelectronic applications.^14,15^

**Figure 2:**
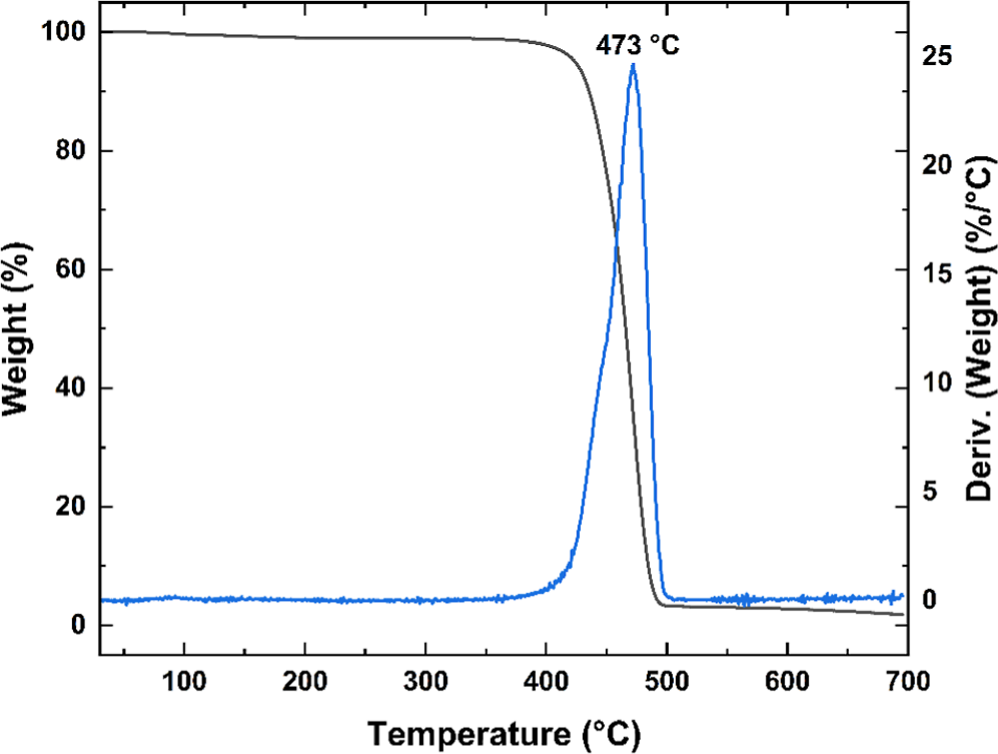
Thermograms and the respective DTG of the synthesized PVK.

In contrast, PVK–Br (Figure 3) exhibited reduced thermal stability, which can be attributed to the presence of reactive bromine substituents. These groups may initiate early thermal degradation through homolytic cleavage of C–Br bonds, leading to the formation of bromine radicals at elevated temperatures. This behavior not only accounts for the lower thermal stability observed but also supports the successful bromination of the polymer backbone.^28^

**Figure 3:**
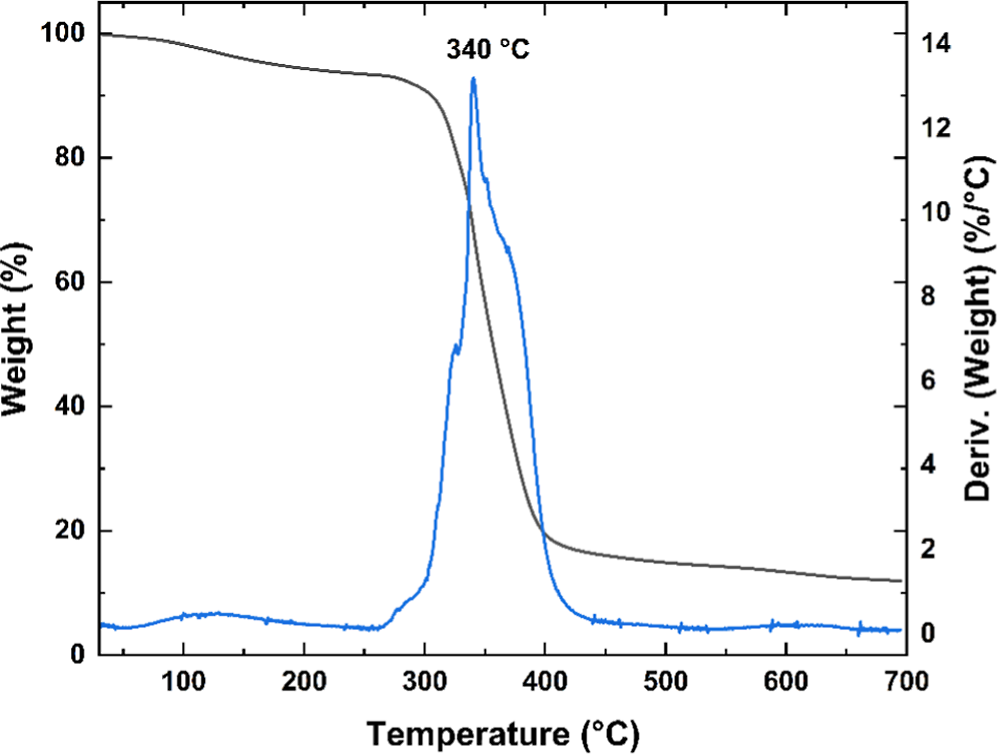
Thermogram and the respective DTG of the synthesized PVK–Br.

The thermogram reveals two distinct mass-loss events. The first is a broad event, centered at approximately 125 °C in the DTG curve and extending up to around 300 °C. This event accounts for approximately 4.7% of the total mass and is likely associated with the initial elimination of bromine radicals. Such thermal debromination processes have been reported for brominated aromatic compounds, where homolytic cleavage of the C–Br bond may occur at relatively low temperatures, generating HBr or brominated volatiles. In the context of PVK–Br, this behavior is consistent with partial thermal decomposition of pendant brominated moieties upon heating.^28–30^

The second and most significant mass-loss event occurs at a peak around 340 °C, representing around 79.2% of the total mass. As mentioned earlier, bromine free radicals can trigger degradation reactions in the polymer backbone. Consequently, this event corresponds to the thermal decomposition of the polymer backbone, which was accelerated by bromine radicals. In addition, bromine elimination can continue above 300 °C, further contributing to this weight loss event and making the calculation PVK’s repetitive unit-to-bromine ratio inaccurate.^30^

Additionally, the introduction of bromine groups can influence the thermal decomposition process by promoting the formation of thermally stable, char-like residues. During degradation, bromine may facilitate cross-linking reactions or stabilize specific polymer fragments, thereby inhibiting their complete volatilization. This behavior accounts for the higher residual mass (16%) observed in PVK–Br, in contrast to pristine PVK, which undergoes nearly complete decomposition. The presence of such cross-linked or stabilized structures enhances the thermal resistance of the residue, leading to more substantial char formation upon thermal treatment. This behavior is consistent with the known degradation pathways of brominated polymers and further corroborates the successful functionalization of PVK with bromine.^28,30,31^

In the case of PVK–An (Figure 4), two distinct weight-loss events are observed. The first, at 149 °C, corresponds to the elimination of unreacted bromine bonds (17.8%), while the second, centered around 398 °C, is attributed to the removal of anthracene side chains (24.8%). These weight losses allow for determination of the PVK’s repetitive unit-to-bromine ratio, and the proportion of anthracene units incorporated into the PVK backbone.^32^

**Figure 4:**
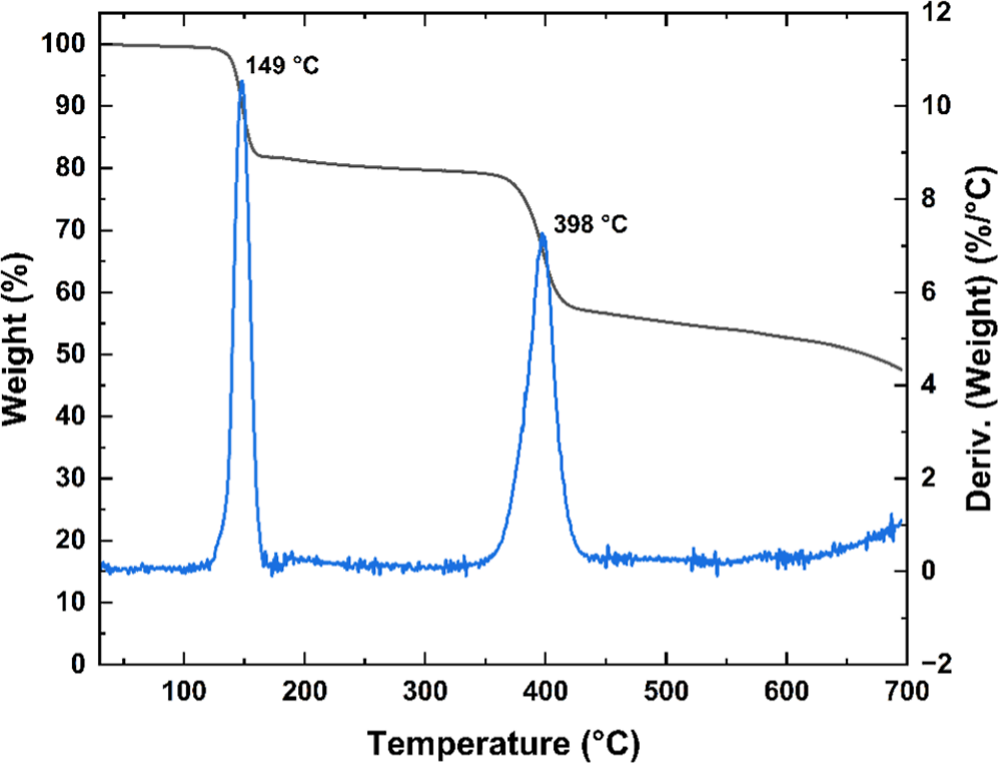
TGA and DTG curves of PVK–An showing two distinct thermal events: the first at 149 °C attributed to the elimination of residual bromine, and the second at 398 °C corresponding to the thermal degradation of the polymer backbone and the loss of anthracene moieties.

Considering the molar masses of bromine (79.9 g·mol^–1^), anthracene (178.23 g·mol^–1^), and the PVK repeat unit (223.2 g·mol^–1^), along with the mass losses observed in the TGA curves (0.459 mg for bromine, 0.640 mg for anthracene, and 1.485 mg total), the molar ratio of PVK to bromine was estimated to be approximately 3:2. This implies that, on average, two bromine atoms are present for every three PVK repeat units. Considering the additional mass loss associated with anthracene and assuming a 1:1 substitution per brominated site, the PVK/anthracene ratio was found to be close to 3:1. Therefore, for every three PVK units, one anthracene moiety was successfully incorporated. These results indicate partial substitution efficiency and are consistent with the presence of residual bromine observed in the thermogram, reflecting an incomplete conversion during the Suzuki coupling step.

This result is reasonable, as the bulky shape of the anthracene units makes it challenging to insert more than one adjacent unit. Instead, it is more favorable to intercalate a nonfunctionalized repeat unit with a functionalized one in the polymer backbone.

The high residual mass exceeding 50%, characteristic of materials rich in condensed aromatic structures, suggests the incorporation of anthracene rigid units onto carbazole backbone. These results collectively confirm the successful incorporation of anthracene groups into PVK, while highlighting that not all carbazole units were functionalized. Furthermore, they emphasize the impact of this functionalization on the polymer’s thermal properties.

### 3.3 UV–Vis Spectroscopy

The UV–vis spectra revealed that all three materials exhibit absorption bands at wavelengths below 340 nm, attributed to the n–π* and π–π* transitions of the carbazole unit, a characteristic feature of blue-emitting materials. This indicates that anthracene functionalization did not alter PVK’s emission properties, preserving its blue emission profile. The analysis was performed on thin films to evaluate the materials in a form more representative of their application in devices.^33–37^

Upon bromination, the absorption spectrum of PVK–Br shows a slight redshift (10 nm), shifting the highest-intensity peak from 235 to 245 nm. This bathochromic shift, characteristic of brominated compounds, suggests the effectiveness of the hydrogen-to-bromine exchange, as can be observed in Figure 5.^38–40^

**Figure 5:**
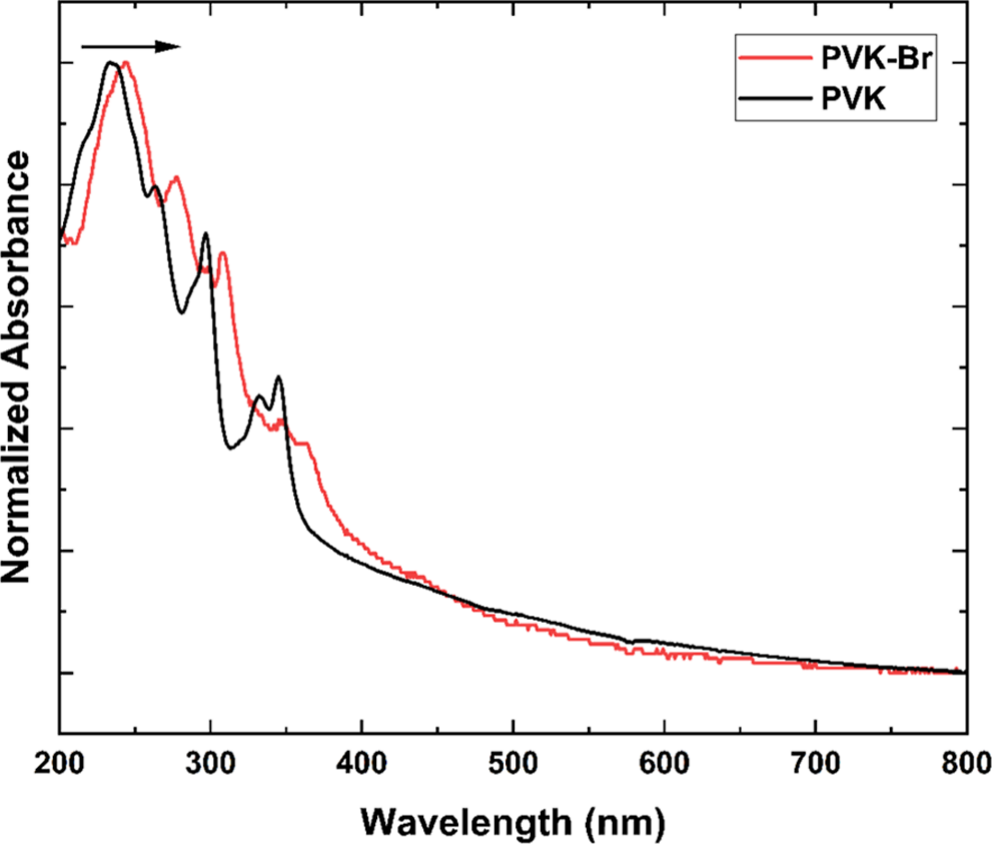
Normalized UV–vis spectra of the synthesized polymers PVK and PVK–Br.

Upon replacement of bromine units with anthracene, the UV spectrum shifts back toward the blue region, with the most intense absorption peak returning to 235 nm, mirroring that of pristine PVK. However, despite this primary blue shift, several absorption bands remain slightly red-shifted relative to the original polymer—specifically, from 265 to 275 nm, 297 to 304 nm, and 345 to 360 nm (Figure 6). These residual red shifts indicate an extended π-conjugation within the polymer backbone, attributed to the incorporation of anthracene’s fused aromatic rings, as well as the presence of residual unfunctionalized bromine moieties that contribute to the overall electronic structure.^41,42^

**Figure 6:**
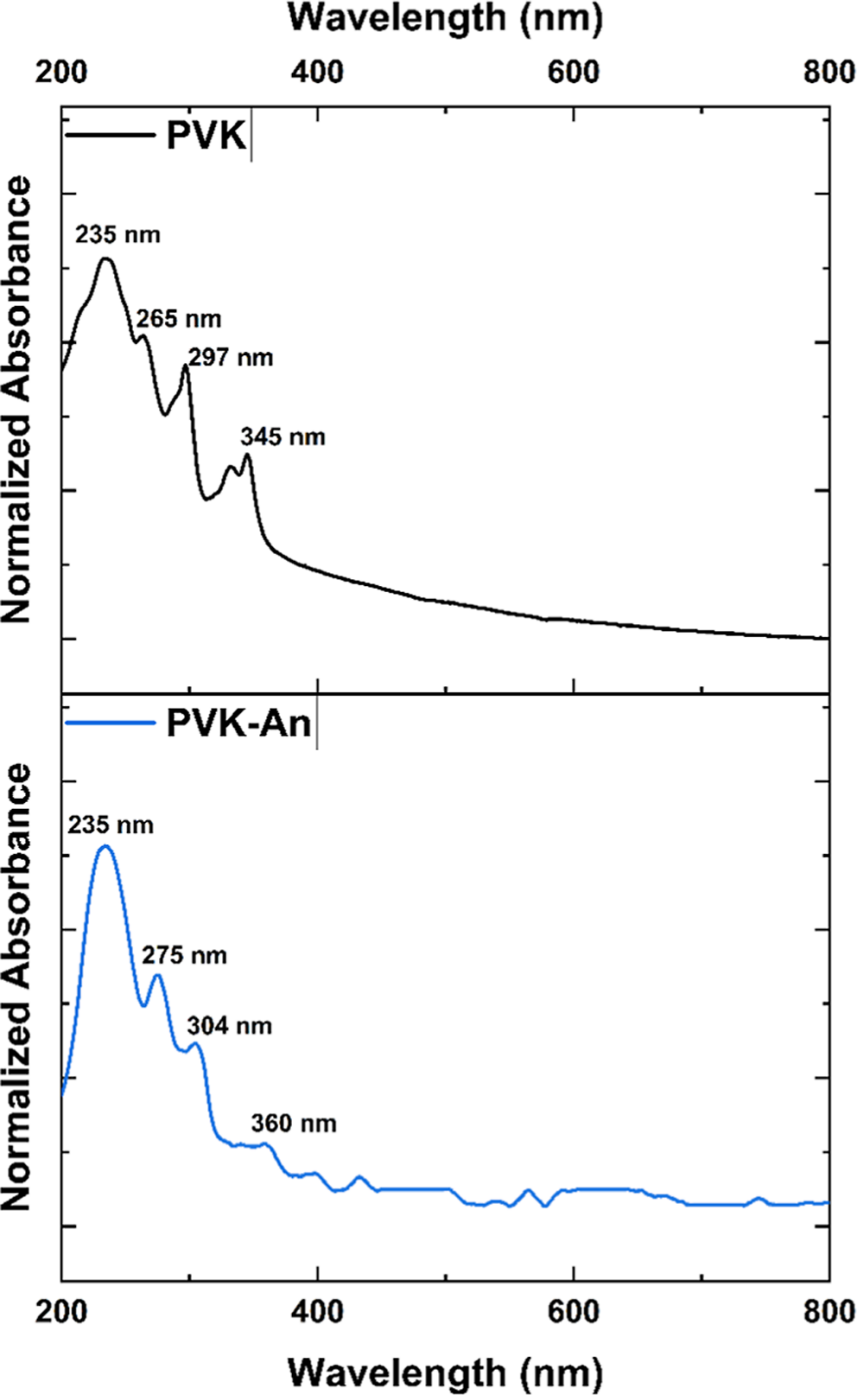
Normalized UV–vis spectra of the synthesized polymers PVK and PVK–An.

### 3.4 Energy Levels

To gain further insight into the electronic properties of the synthesized polymers, the HOMO and LUMO energy levels were estimated based on the onset of UV–vis absorption and photoluminescence emission maxima. These values are crucial for evaluating the materials’ suitability in optoelectronic applications, particularly their ability to facilitate charge injection and transport. Table 2 summarizes the calculated optical bandgaps and energy level positions for PVK, PVK–Br, and PVK–An.

**Table 2:** UV–Vis Absorption Onset (λ_onset_) and Calculated Optical Bandgap (*E*_g_^opt^) for the Synthesized Polymers

polymer	λ_onset_ (nm)	*E*_g_^opt^ (eV)
PVK	363	3.42
PVK–Br	423	2.93
PVK–Func	380	3.26

The calculated optical bandgap values align with the results observed in the UV–vis spectra. All polymers exhibited a high bandgap, close to 3 eV, which is characteristic of blue-emitting materials. This further confirms that the functionalization with anthracene did not alter the emission region of PVK, maintaining its blue emission profile.^43–45^

Additionally, the data is consistent with the slight redshift observed for PVK–Br in the UV–vis analysis. This redshift is reflected in the lower bandgap of 2.93 eV for PVK–Br, which is characteristic of brominated materials. These findings corroborate with successful bromination.

Figure 7 illustrates the energy band diagram of the synthesized polymers, highlighting their respective HOMO and LUMO energy levels. In the case of PVK–An, the incorporation of anthracene likely introduced extended conjugation or electron-withdrawing effects contributing to the observed reduction in the LUMO level.^46,47^ This is a key factor to consider, particularly in the context of OLED device performance. A lower LUMO level facilitates electron injection from the cathode into the emissive layer, thereby reducing the energy barrier for charge carrier injection. This enhancement improves charge balance within the emissive layer, which is essential for optimizing device efficiency and stability.^48,49^

**Figure 7:**
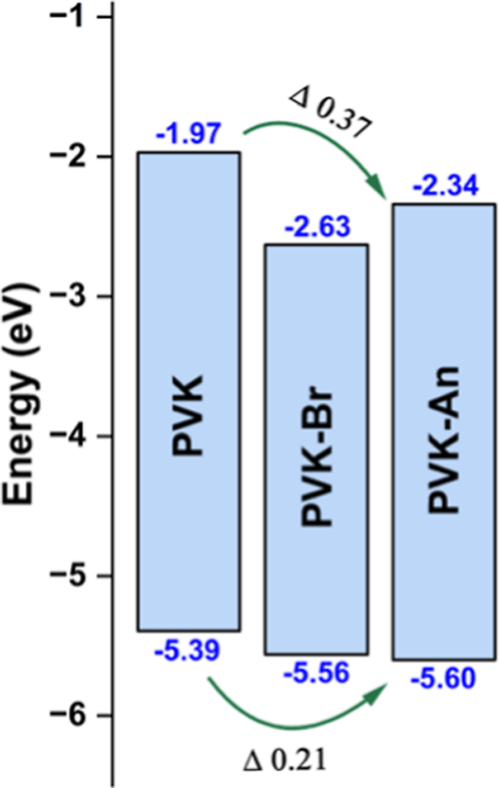
Energy band diagram of the synthesized polymers showing experimental HOMO and LUMO values determined by cyclic voltammetry (Films on ITO—WE, Ag/AgCl—RE, platinum rod—CE, electrolytic solution: TBAPF6 in acetonitrile (0.1 mol·dm^–3^); potential range ±2 mV, scan rate 50 mV•s^–1^) and UV–vis spectroscopy (films on quartz substrates, 200–800 nm).

Regarding the HOMO level, functionalization with anthracene increases the oxidation potential to 1.20 eV, resulting in a HOMO level of −5.60 eV, which indicates greater stability (Figure 8). This shift aligns with the stabilization of the LUMO energy levels, attributed to the extended conjugation introduced by the anthracene units.

**Figure 8:**
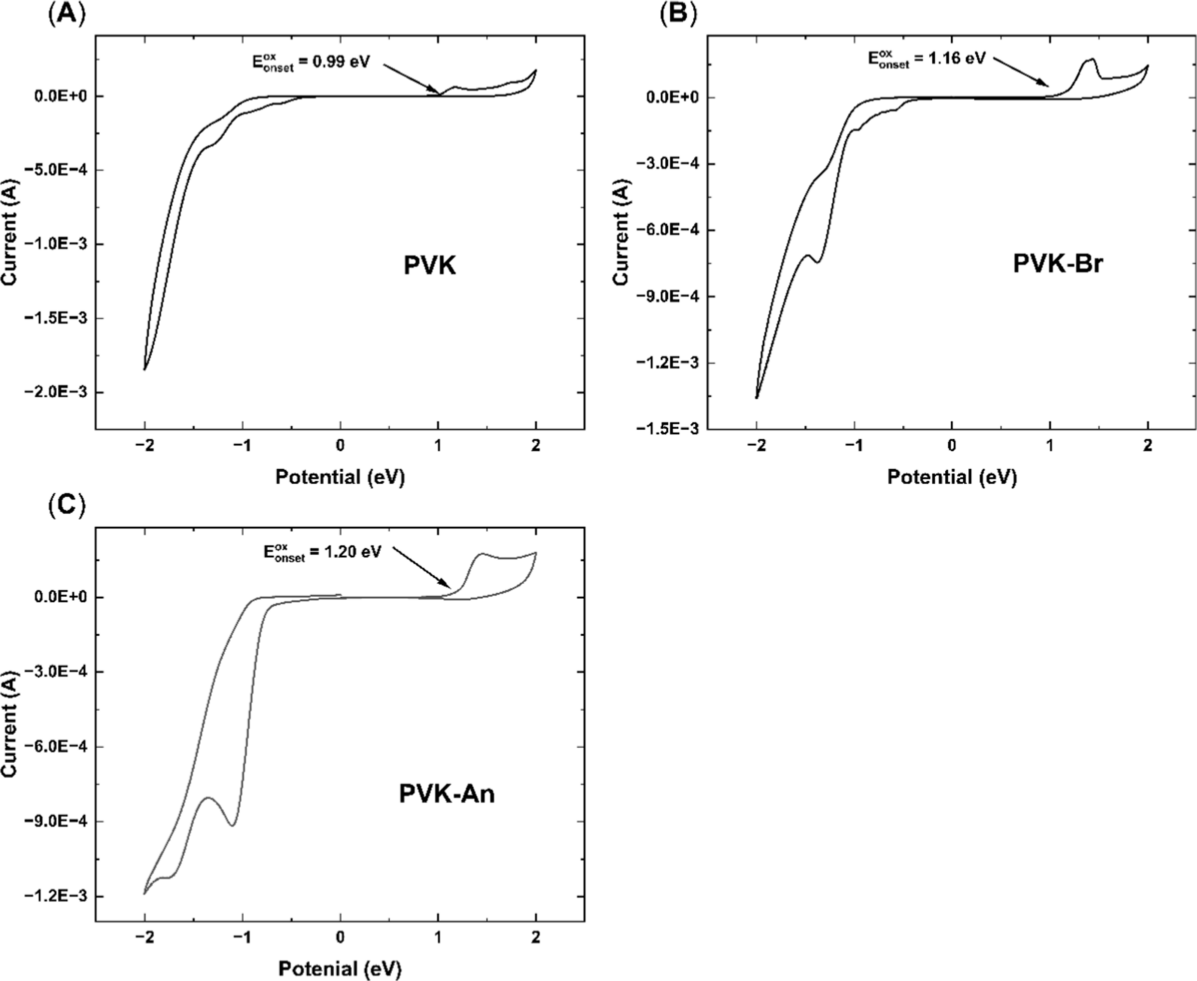
Voltammograms of the synthesized polymers. (A) PVK; (B) PVK–Br and (C) PVK–An.

The shift in the LUMO level without altering the blue emission profile of PVK highlights the dual benefit of the functionalization process: maintaining the desired optical properties while improving electronic properties critical for OLED functionality. This balance is a key factor in designing materials tailored for efficient optoelectronic devices.

### 3.5 Photoluminescence (PL) Spectroscopy

The fluorescence of the pure materials was initially investigated, and their emission spectra revealed that all polymers exhibited blue emission with wavelengths below 435 nm, as shown in Figure 9A. An 18 nm red shift was observed for PVK–An compared to pure PVK. This shift does not alter the characteristic blue emission of PVK, instead, it moves further into the visible spectrum, which is highly desirable for OLED applications, Figure 9B. As expected, the photoluminescence intensity of the neat films was low. In the case of PVK–Br, the presence of bromine likely contributes to fluorescence quenching, as bromine atoms can act as heavy atom quenchers, promoting intersystem crossing (ISC) and thereby reducing photoluminescence. This behavior agrees with previously reported trends in halogenated polycarbazoles.^50–52^ In the case of PVK–An, the polymer’s high content of aromatic rings, due to the introduction of anthracene units, enhances intermolecular π–π interactions, leading to an aggregation-caused quenching (ACQ) effect that drastically decreases emission intensity.^53,54^

**Figure 9:**
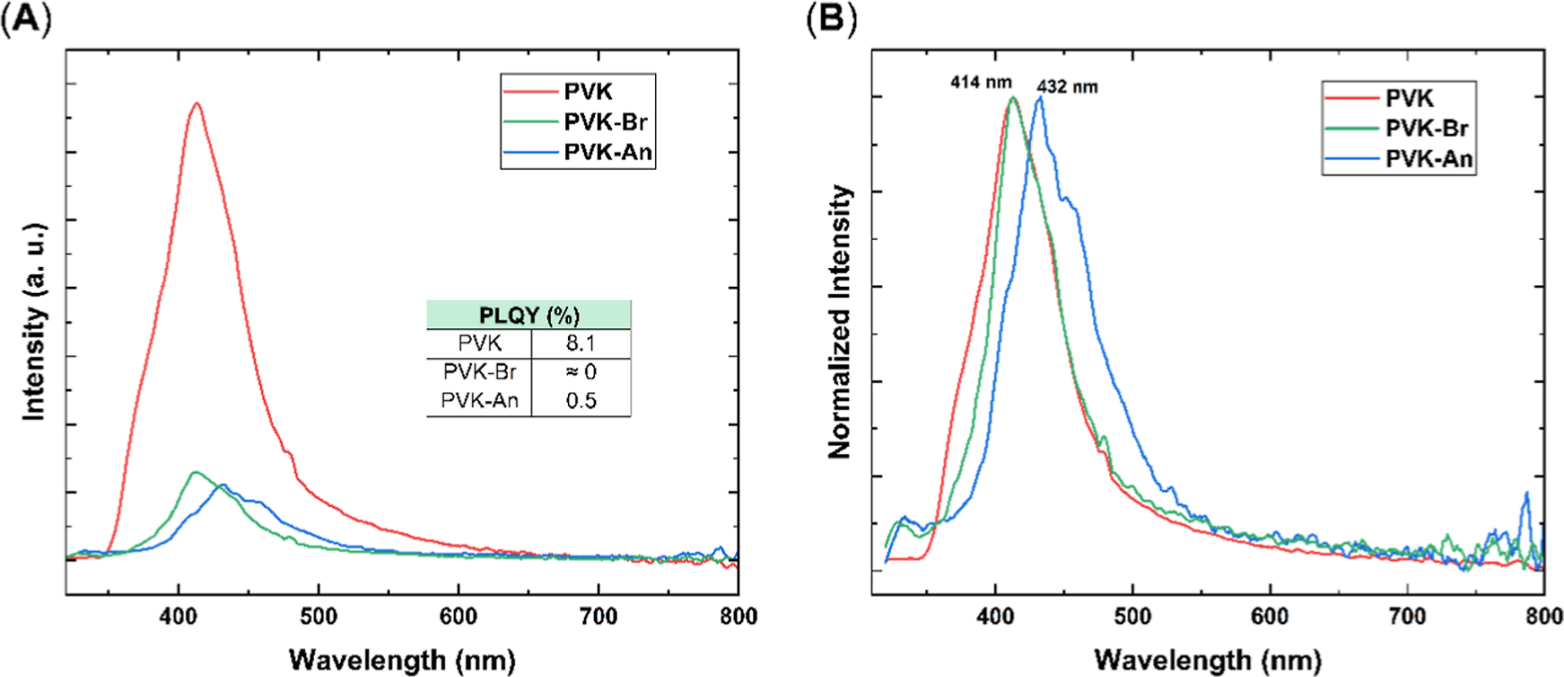
Photoluminescence spectra of the synthesized polymers in thin-film λ_ex_ = 300 nm for PVK and PVK–An and λ_ex_ = 330 nm for PVK–Br. (A) Non-normalized spectra with the respectively PLQYs. (B) Normalized spectra.

The photoluminescence quantum yield (PLQY) data supports these observations, following the same trend: PVK–Br exhibits an almost negligible PLQY of approximately 0%, PVK–An shows a slightly higher PLQY of 0.5% and pure PVK displays the higher PLQY of around 8%, indicating its increased radiative decay.

To address the aggregation-caused quenching (ACQ) effect, the synthesized PVK–An polymer was incorporated into an inert PMMA matrix, enabling solid-state dilution of the conjugation-rich polymer chains.^53,54^ This approach aimed to spatially separate the emissive units and minimize intermolecular interactions that typically lead to nonradiative decay in the solid state. As a result, a remarkable improvement in photoluminescence quantum yield (PLQY) was observed, with a nearly 58-fold increase—reaching 28.9%, as shown in Figure 10. These findings confirm that the reduced emission efficiency of neat PVK–An films was primarily attributed to ACQ, which was intensified by the extended π-conjugation of the anthracene units. By suppressing this effect through dilution in PMMA, the intrinsic emission potential of PVK–An could be effectively restored. The substantial improvement in PLQY underscores the potential of PVK–An/PMMA composites as emissive layers in OLED devices, combining the desirable optoelectronic properties of anthracene with the mechanical and morphological stability provided by the host matrix.

**Figure 10:**
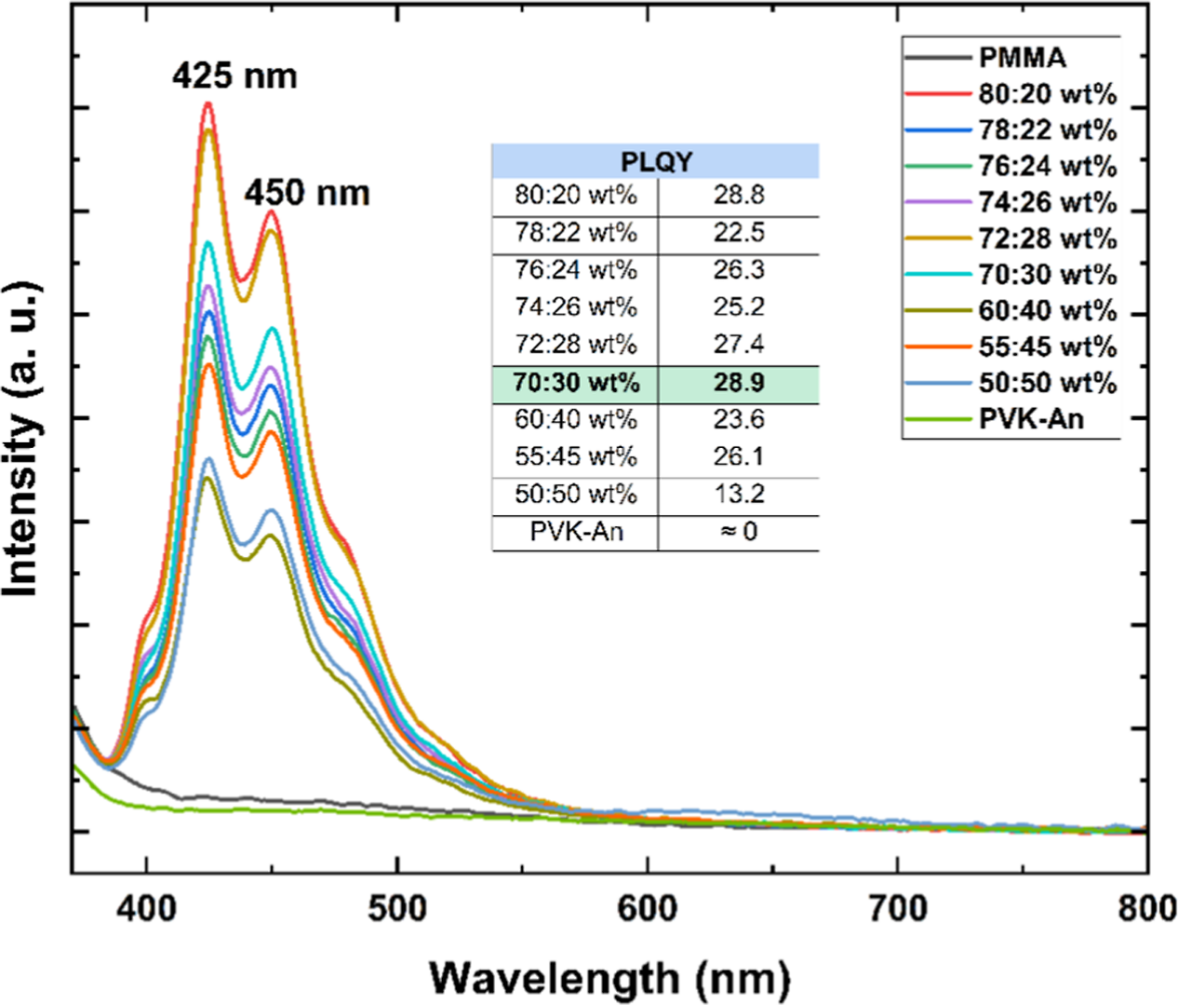
Photoluminescence spectra of the PMMA/PVK–An matrices in different ratios λ_ex_ = 290 nm, recorded on the integrating sphere and their respective PLQY values.

The more intense spectra obtained after solid-state dilution revealed a distinct emission profile for PVK–An compared to unmodified PVK. The PVK–An spectrum now exhibits two peaks: one at 425 nm, close to the PVK emission at 410 nm, and another at 450 nm. The red shift observed in the neat films was preserved in the matrix spectra, supporting the hypothesis that increased conjugation is responsible for the emission at longer wavelengths. Furthermore, the excitation spectra confirmed that both peaks originate from a single emitting species (Figure 11), suggesting a new radiative decay process within the polymer core. This process may be associated with structural changes induced by the introduction of the anthracene unit, previously undetectable due to weak emission intensity in neat films.^55–57^

**Figure 11:**
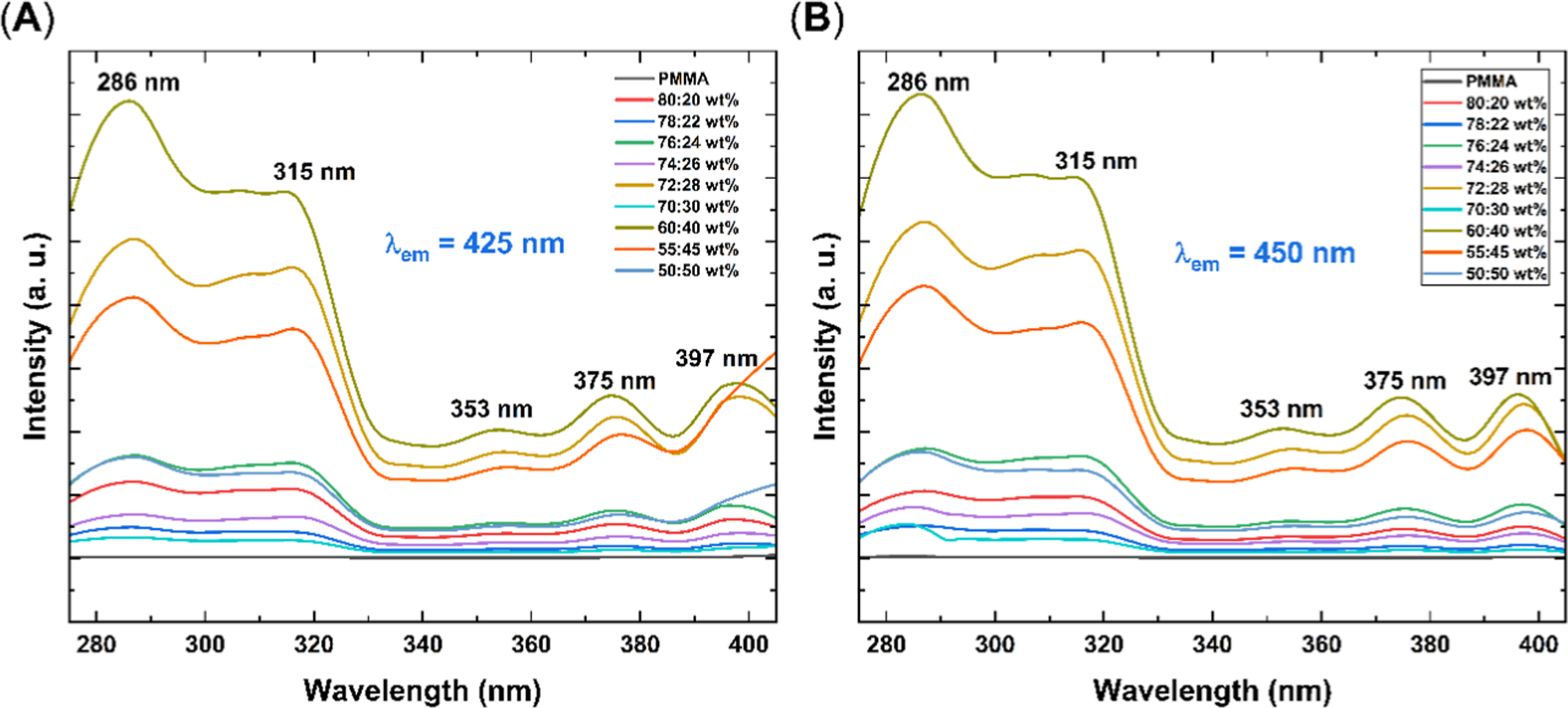
Excitation spectra of the PMMA/PVK–An matrices in different ratios. (A) Emission at 425 nm. (B) Emission at 450 nm.

Time resolved photoluminescence (TRPL) measurements were carried out for both emission bands, and the results revealed two decay components with comparable lifetimes (Table 3). This similarity in decay times diminishes the probability that the second emission arises from a distinct charge transfer (CT) state. Instead, the data suggest that both emissions likely originate from the same singlet excited state (S_1_), with the second band corresponding to radiative transitions from different vibrational sublevels or conformational states within the S_1_ → S_0_ manifold. This interpretation is consistent with the vibronic nature of emissions observed in conjugated systems.^57–60^

**Table 3:** Fluorescence Lifetime Results of the PMMA/PVK–An 70:30 wt % Matrix Recorded on Thin Film

λ_em_	τ_1_ (ns)	Rel. (%)	τ_2_ (ns)	Rel. (%)	χ^2^
425	2.71 ± 0.18	27.68	12.32 ± 0.38	73.32	1.14
450	2.90 ± 0.19	26.24	13.69 ± 0.41	73.76	0.98

To further assess the role of the anthracene unit in modulating the emissive behavior of PVK, control experiments were conducted using unmodified PVK dispersed in a PMMA matrix. This approach aimed to isolate the effects of aggregation on its photoluminescence efficiency. Upon dilution, only a modest increase in PLQY was observed, with the emission reaching 10.9%—representing a 1.3-fold enhancement compared to the neat PVK film (Figure 12). These findings suggest that aggregation has a limited impact on the emission efficiency of PVK itself. In contrast, the substantial PLQY enhancement observed for PVK–An in the same matrix underscores the critical role of the anthracene moiety in promoting radiative recombination. This result highlights the potential of anthracene-functionalized PVK as a promising emissive material, where the chromophore contributes actively to improving luminescence upon suppression of quenching mechanisms.

**Figure 12:**
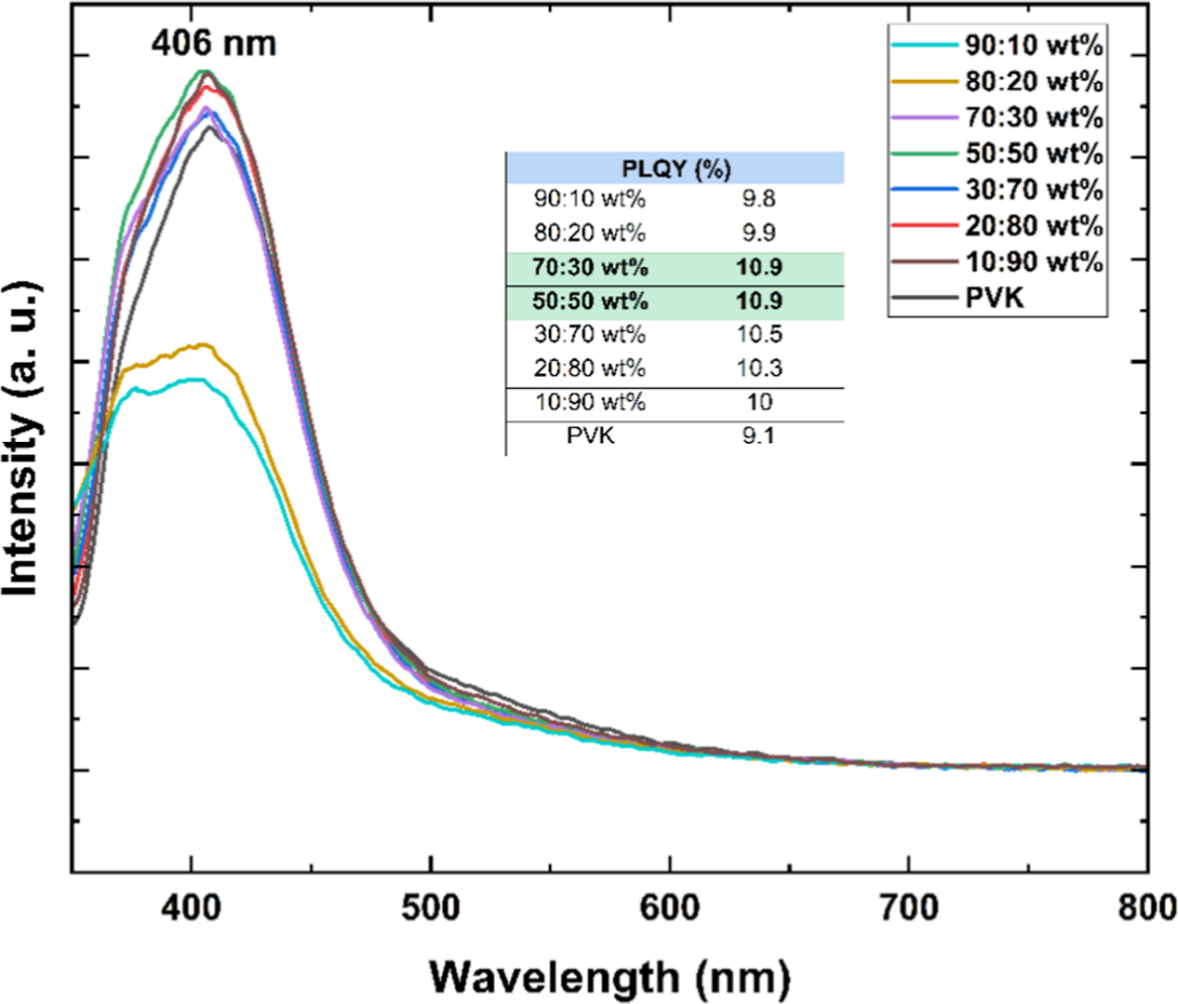
Emission spectra of PMMA/PVK matrices recorded on the integrating sphere.

### 3.6 Process of Fluorescence Decay

The time-resolved photoluminescence data reveal biexponential decay behavior for all samples, which is indicative of multiple emissive processes occurring within the excited state. This dual decay can be attributed to a combination of localized singlet exciton emission and intermolecular interactions such as excimer formation. The results are summarized in Table 4.

**Table 4:** Fluorescence Lifetime Results of the Synthesized Polymers Recorded on Thin Film

polymer	τ_1_ (ns)	Rel. (%)	τ_2_ (ns)	Rel. (%)	χ^2^
PVK	6.16 ± 0.15	27.67	25.08 ± 0.26	72.33	1.19
PVK–Br	0.64 ± 0.02	57.32	10.48 ± 0.45	42.68	1.20
PVK–An	4.75 ± 0.18	45.20	15.42 ± 0.60	54.80	1.15
PMMA/PVK–An 70:30 wt %	5.50 ± 0.26	32.14	17.28 ± 0.60	67.87	1.09

In the case of pristine PVK, the dominant long-lived component (τ_2_ = 25.08 ns) strongly suggests the presence of excimers—excited-state dimers typically formed through π–π stacking of carbazole units.^61,62^ These excimer states emit at lower energy and with reduced efficiency due to their delocalized, which may contribute to the limited emissive properties of PVK.^63–66^

In contrast, PVK–Br exhibits a pronounced reduction in both lifetimes, especially τ_1_ = 0.64 ns, reflecting strong intersystem crossing (ISC) facilitated by the heavy atom effect of bromine, which supports the findings observed in the PL measurements.^67–70^

For PVK–An, the introduction of anthracene units leads to intermediate lifetimes (τ_2_ = 15.42 ns) and a diminished contribution from the longer decay component, suggesting a reduced excimer formation. This behavior is likely due to steric hindrance and electronic decoupling between polymer chains introduced by the anthracene moieties. Supporting this hypothesis, the PMMA/PVK–An 70:30 wt % matrix exhibits behavior similar to that of the neat PVK–An film, indicating that the pure polymer effectively limits excimer formation. Furthermore, the longer decay time compared to PVK–Br suggests a lower degree of quenching.

To illustrate the distinct emissive behaviors of the synthesized polymers, a simplified decay mechanism diagram is proposed (Figure 13). Upon excitation, the system reaches the first excited singlet state (S_1_). In the case of PVK–Br, ISC facilitated by the heavy atom effect of bromine promotes the transition from S_1_ to the triplet state (T_1_), leading predominantly to nonradiative decay and resulting in the negligible emission observed in the PL measurements. For unmodified PVK, emission arises primarily from excimer states (S_1_*), which decay radiatively to the ground state (S_0_) with a longer lifetime of approximately 25 ns (Rel. 72.3%). In contrast, PVK–An exhibits emission from both the S_1_ → S_0_ and S_1_* → S_0_ transitions with similar contributions. Although excimer formation is still present (τ_2_ ≈ 15 ns), its reduced contribution to the overall emission indicates a more efficient radiative decay pathway in the modified polymer, reflecting enhanced emissive performance.

**Figure 13:**
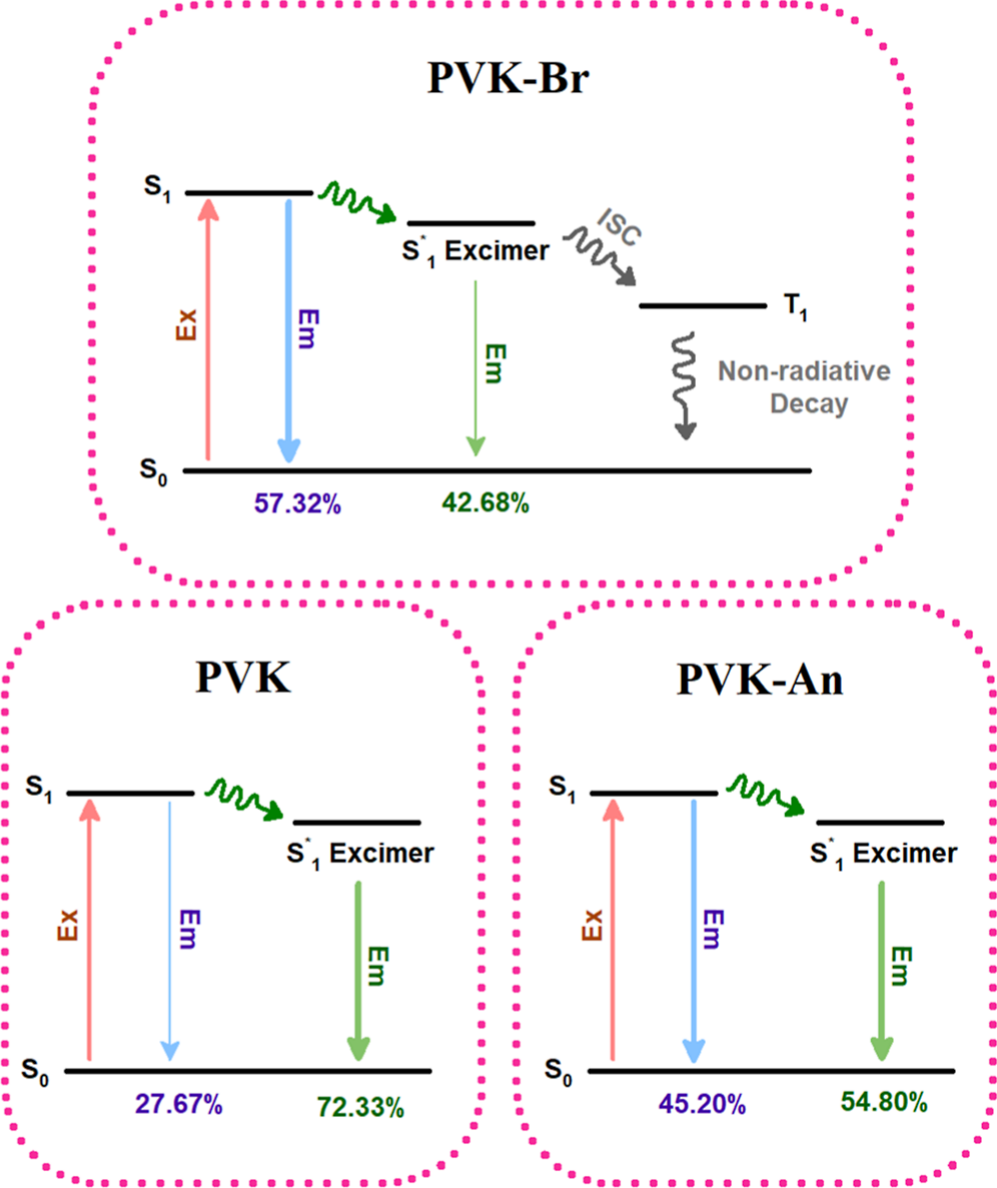
Qualitative diagram of electron dynamics and radiative/nonradiative decay pathways in PVK–based polymers. Ex—excitation, Em—emission, green wavy arrow—internal conversion and ISC—intersystem crossing.

These findings underscore the importance of side-chain engineering and matrix selection in tuning the balance between radiative and nonradiative decay channels in emissive polymers for OLED applications.

## 4 Conclusions

In this study, PVK was successfully synthesized and functionalized with anthracene moieties through a sequential strategy, as confirmed by comprehensive structural (^1^H NMR), thermal (TGA), and optoelectronic characterizations. Structural and thermal analyses indicate that anthracene incorporated into the PVK backbone at an approximate ratio of 3:1 PVK repeat units to anthracene.

The initial photophysical results showed that the functionalization preserved the intrinsic blue emission of PVK. Although photoluminescence quenching was initially observed in PVK–An, attributed to aggregation effects and π–π interactions, these limitations were effectively mitigated by incorporating the material into an inert PMMA matrix. This strategy led to a substantial increase in PLQY, from 0.5% in neat PVK–An to 28.9% in the optimized 70:30 wt % PMMA/PVK–An blend. Interestingly, applying the same dilution strategy to pristine PVK did not enhance its photoluminescence properties, suggesting that PVK does not suffer from ACQ and further highlights the improved emissive behavior achieved through functionalization.

Time-resolved photoluminescence revealed distinct emission mechanisms among the PVK derivatives. Neat PVK showed significant excimer formation, which was the primary contributor to its emission (Rel. 72.33%). In contrast, PVK–An exhibited reduced excimer formation, with a lower contribution to the overall emission (Rel. 54.80%), consistent with its enhanced emissive efficiency.

Taken together, these findings underscore the dual benefits of side-chain engineering (via anthracene functionalization) and morphological control (via PMMA dispersion) for achieving high-efficiency blue-emitting materials. While this work primarily focuses on photophysical and thermal characterization, it lays a robust foundation for future integration into OLED devices. Follow-up studies should explore exciton transport, device fabrication, and emission stability under electrical bias to further validate these materials for advanced optoelectronic applications.

## Supplementary Material



## Notes

The ^1^H NMR spectrum zoom-viewed comparing PVK and PVK–An, the absorption spectrum of PVK–Br (unnormalized), excitation and emission spectra of the neat films at different wavelengths, as well as the spectra of PMMA/PVK blends at various ratios and wavelengths. Additionally, it provides the corresponding decay curves for each sample, including the optimized PMMA/PVK–An matrix (PDF)

## References

[1] Liguori R., Nunziata F., Aprano S., Maglione M. G. (2024). Overcoming Challenges
in OLED Technology for Lighting Solutions. Electronics.

[2] Bhattarai T., Ebong A., Raja M. Y. A. (2024). A Review of Light-Emitting
Diodes
and Ultraviolet Light-Emitting Diodes and Their Applications. Photonics.

[3] Potoczny, G. A. Roll-to-roll Production Challenges for Large-area Printed Electronics. Flexible Flat Panel Displays; Wiley, 2023; pp 325–336.

[4] Zeng X. Y., Tang Y. Q., Cai X. Y., Tang J. X., Li Y. Q. (2023). Solution-Processed
OLEDs for Printing Displays. Mater. Chem. Front..

[5] Woo J. Y., Park M.-H., Jeong S.-H., Kim Y.-H., Kim B., Lee T.-W., Han T.-H. (2023). Advances in Solution-Processed OLEDs
and Their Prospects for Use in Displays. Adv.
Mater..

[6] de
Brito E. B., Valaski R., Marques M. d. F. V. (2020). Development
of Polymeric Active Layer for RGB Light-Emitting Devices: A Review. J. Mater. Sci.: Mater. Electron..

[7] Wang L., Chen T., Lin Q., Shen H., Wang A., Wang H., Li C., Li L. S. (2016). High-Performance
Azure Blue Quantum Dot Light-Emitting Diodes via Doping PVK in Emitting
Layer. Org. Electron..

[8] Zhang L., Hu D., Wang S., Ma Y. (2024). High-Performance
Solution-Processed
Blue OLEDs Based on “Hot Exciton” Materials. J. Lumin..

[9] Sun N., Zou Q., Chen W., Zheng Y., Sun K., Li C., Han Y., Bai L., Wei C., Lin J., Yin C., Wang J., Huang W. (2023). Fluorene Pendant-Functionalization
of Poly­(N-Vinylcarbazole) as Deep-Blue Fluorescent and Host Materials
for Polymer Light-Emitting Diodes. Chin. Chem.
Lett..

[10] Kagatikar S., Sunil D. (2022). A Systematic Review
on 1,8-Naphthalimide Derivatives as Emissive
Materials in Organic Light-Emitting Diodes. J. Mater. Sci..

[11] Corrêa
Santos D., Vieira Marques M. F. (2022). Blue Light Polymeric Emitters for
the Development of OLED Devices. J. Mater. Sci.:
Mater. Electron..

[12] Zhang Z., Jiang W., Ban X., Yang M., Ye S., Huang B., Sun Y. (2015). Solution-Processed Efficient Deep-Blue
Fluorescent Organic Light-Emitting Diodes Based on Novel 9,10-Diphenyl-Anthracene
Derivatives. RSC Adv..

[13] Yang X., Xu X., Zhou G. (2015). Recent Advances of the Emitters for High Performance
Deep-Blue Organic Light-Emitting Diodes. J.
Mater. Chem. C.

[14] Mota I. C., Marques M. F. V. (2019). Synthesis of
Polyvinylcarbazole/Reduced Graphite Oxide-ZnO
Nanocomposites. Macromol. Symp..

[15] Barbosa
de Brito E., de Morais A., Nei de Freitas J., Valaski R., de Fátima Vieira Marques M. (2022). Improved Properties
of High Molar Mass Poly­(9-Vinylcarbazole) and Performance as a Light
Emitter Compared with the Commercial PVK. Mater.
Sci. Eng. B.

[16] Geffroy C., Grana E., Mumtaz M., Cojocaru L., Cloutet E., Olivier C., Uchida S., Toupance T., Segawa H., Hadziioannou G. (2019). Post-Functionalization
of Polyvinylcarbazoles: An Open
Route towards Hole Transporting Materials for Perovskite Solar Cells. Sol. Energy.

[17] Moreira C. M. S., Marques M., Viana L. M., Mothé M. G., Santos D. C. (2024). Synthesis and Characterization of
Functionalized Poly­(9-Vinylcarbazole)
for Light-Emitting Diode Applications. Obs.
Econ. Latinoam..

[18] Rubio
Arias J. J., Mota I. C., Vieira Marques M. D. F. (2021). Synthesis
of Thiophene-Benzodithiophene Wide Bandgap Polymer and GIWAXS Evaluation
of Thermal Annealing with Potential for Application in Ternary Polymer
Solar Cells. Polym. Adv. Technol..

[19] Casey A., Dimitrov S. D., Shakya-Tuladhar P., Fei Z., Nguyen M., Han Y., Anthopoulos T. D., Durrant J. R., Heeney M. (2016). Effect of
Systematically Tuning Conjugated Donor Polymer Lowest Unoccupied Molecular
Orbital Levels via Cyano Substitution on Organic Photovoltaic Device
Performance. Chem. Mater..

[20] Köhler, A. ; Bässler, H. Electronic Processes in Organic; Wiley-VCH; John Wiley [distributor], 2015.

[21] Otterbach S. A., Elsing D., Schulz A. D., Tappert H., Wenzel W., Kozlowska M., Röhm H., Bräse S. (2024). Pseudo-Para-Substituted
[2.2]­Paracyclophanes for Hole Transport in Perovskite Solar Cells. Adv. Funct. Mater..

[22] Sworakowski J. (2018). How Accurate
Are Energies of HOMO and LUMO Levels in Small-Molecule Organic Semiconductors
Determined from Cyclic Voltammetry or Optical Spectroscopy?. Synth. Met..

[23] Wang J., Leung L. M., So S. K., Chan C. Y. (2014). Blue Fluorescent
Conductive Poly­(9,10-Di­(1-Naphthalenyl)-2-Vinylanthracene) Homopolymer
and Its Highly Soluble Copolymers with Styrene or 9-Vinylcarbazole. Polym. Int..

[24] Santos D. C., de Paula T. P., de Brito E. B., Arias J. J. R., Vieira
Marques M. d. F. (2022). Influence of Reaction Conditions on Kumada Catalytic
Transfer Polymerization for Synthesis of Poly­(p-Phenylene) for Organic
Semiconductors. J. Polym. Res..

[25] Fulmer G. R., Miller A. J. M., Sherden N. H., Gottlieb H. E., Nudelman A., Stoltz B. M., Bercaw J. E., Goldberg K. I. (2010). NMR Chemical
Shifts
of Trace Impurities: Common Laboratory Solvents, Organics, and Gases
in Deuterated Solvents Relevant to the Organometallic Chemist. Organometallics.

[26] Gottlieb H. E., Kotlyar V., Nudelman A. (1997). NMR Chemical Shifts of Common Laboratory
Solvents as Trace Impurities. J. Org. Chem..

[27] Tienne L. G. P., Paula T. P., de Fátima
Vieira Marques M., Wedel A., Nogueira A. F. (2025). Unveiling the Impact
of Downscale
Reactions on P3HT Synthesis: A Comprehensive Exploration of Properties
and Photovoltaic Device Performance. Polym.
Adv. Technol..

[28] Beach M. W., Hull J. W., King B. A., Beulich I. I., Stobby B. G., Kram S. L., Gorman D. B. (2017). Development
of a New Class of Brominated
Polymeric Flame Retardants Based on Copolymers of Styrene and Polybutadiene. Polym. Degrad. Stab..

[29] Lin K., Zhen S., Ming S., Xu J., Lu B. (2015). Synthesis
and Electro-Optical Properties of New Conjugated Hybrid Polymers from
EDOT End-Capped Dibenzothiophene and Dibenzofuran. New J. Chem..

[30] Bromberg L., Pomerantz N., Schreuder-Gibson H., Hatton T. A. (2014). Degradation of Chemical
Threats by Brominated Polymer Networks. Ind.
Eng. Chem. Res..

[31] An H., Lee A. S., Kammakakam I., Sang Hwang S., Kim J. H., Lee J. H., Suk Lee J. (2018). Bromination/Debromination-Induced
Thermal Crosslinking of 6FDA-Durene for Aggressive Gas Separations. J. Membr. Sci..

[32] Cataldo F., García-Hernández D. A., Manchado A. (2014). Sonochemical Synthesis
of Fullerene C 60/Anthracene Diels-Alder Mono and Bis-Adducts. Fullerenes, Nanotubes Carbon Nanostruct..

[33] Li L., Hu T.-Q., Yin C.-R., Xie L.-H., Yang Y., Wang C., Lin J.-Y., Yi M.-D., Ye S.-H., Huang W. (2015). A Photo-Stable and
Electrochemically Stable Poly­(Dumbbell-Shaped
Molecules) for Blue Electrophosphorescent Host Materials. Polym. Chem..

[34] Chen, Y. ; Cai, R.-F. ; Huang, Z.-E. ; Bai, X. ; Yu, B.-C. ; Jin, W. ; Pan, D.-C. ; Wang, S.-T. Researches on the Photoconductivity and UV-Visible Absorption Spectra of the First C6o-Chemically Modified Poly(N-Vinylcarbazole); Springer-Verlag, 1996; Vol. 36.

[35] Lange A., Flügge H., Fischer B., Schmidt H., Boeffel C., Wegener M., Riedl T., Kowalsky W. (2012). Optoelectronic Devices
Based on Ultra-Violet Light Sensitive PVK:PCBM Layers. Synth. Met..

[36] Rivaton A., Mailhot B., Derderian G., Bussiere P. O., Gardette J. L. (2003). Investigation
of the Photophysical Processes and Photochemical Reactions Involved
in PVK Films Irradiated at λ > 300 Nm. Macromolecules.

[37] Sonone R. S., Raut V. M., Murhekar G. H. (2014). Structural and Electroluminescence
Properties of Pure PVK and Doped Tio_2_ Polymer Thin Films. Int. J. Adv. Res. Chem. Sci..

[38] Nussbaum A. L., Mancera O., Daniels R., Rosenkranz G., Djerassi C. (1951). The Effect of Bromine Substitution upon the Ultraviolet
Absorption Spectra of α,β-Unsaturated Ketones. J. Am. Chem. Soc..

[39] Bendig P., Vetter W. (2013). UV-Induced Formation of Bromophenols from Polybrominated
Diphenyl Ethers. Environ. Sci. Technol..

[40] Sanetra J., Armatys P., Chrzaszcz R., Pielichowski J., Barta P., Niziol S., Saliraoui B. (1999). Synthesis
and Luminescent Properties of Br-Substituted Poly­(N-Vinylcarbazoles). Synth. Met..

[41] Vacareanu L., Bejan A. E., Bejan D., Pascariu P., Damaceanu M. D. (2025). Design,
Synthesis and Characterization of Triphenylamine-Based Conjugated
Porous Polymers as Fluorescent Receptors for Nitroaromatic Derivatives. Dyes Pigm..

[42] Mo D., Zhang J., Deng K., Chao P. (2025). Unraveling the Monomer
Conjugation Length Effect on the Optoelectronic Performances of Thiophene-EDOT
Hybrid Electrochromic Polymers. Polymer.

[43] Gupta N., Nagar M. R., Anamika, Gautam P., Maiti B., Jou J. H., Kuila B. K. (2023). Triazine and Thiophene-Containing
Conjugated Polymer
Network Emitter-Based Solution-Processable Stable Blue Organic LEDs. ACS Appl. Polym. Mater..

[44] Cheng X., Zhu Y., Li L., Basit A., Zaman N., Chen X., Xue Q., Xu X., Xu G. (2025). Efficient Full-Color Quantum Dot-Based
Light-Emitting Diodes via Hybrid Polymer-Tailored Hole Transport Engineering. J. Phys. Chem. C.

[45] Saad A., Hamad N., Redoy R. A. F., Zhao S., Wageh S. (2024). Enhancing
Blue Polymer Light-Emitting Diode Performance by Optimizing the Layer
Thickness and the Insertion of a Hole-Transporting Layer. Polymers.

[46] Dong J., Zhao C., Ning J., Liu Y., Dou X. (2025). Noncovalent
Interaction-Based Probe Design for PET-Facilitated Fluorescence Sensing
of Synthetic Cannabinoids. ACS Omega.

[47] Long X., Dou C., Liu J., Wang L. (2017). Fine-Tuning
LUMO Energy Levels of
Conjugated Polymers Containing a B ←n Unit. Macromolecules.

[48] Li M., Li W., Zhou J., Tian X., Li H., Jiang Z., Liu D., Liu Y., Wang Y., Shi Y. (2025). N-Oxide-Functionalized
Bipyridines as Strong Electron-Deficient Units to Construct High-Performance
n-Type Conjugated Polymers. Advanced Science.

[49] Lee, J.-H. ; Kim, J.-J. Interfacial Doping for Efficient Charge Injection in Organic Semiconductors. Physics of Organic Semiconductors; Brutting, W. , Adachi, C. , Eds.; Wiley, 2012.

[50] Huang T., Song X., Cai M., Zhang D., Duan L. (2021). Improving
Reverse Intersystem Crossing in Exciplex-Forming Hosts by Introducing
Heavy Atom Effect. Mater. Today Energy.

[51] Kirillova T. N., Gerasimova M. A., Nemtseva E. V., Kudryasheva N. S. (2011). Effect
of Halogenated Fluorescent Compounds on Bioluminescent Reactions. Anal. Bioanal. Chem..

[52] Malinge A., Kumar S., Chen D., Zysman-Colman E., Kéna-Cohen S. (2024). Heavy Atom Effect in Halogenated MCP and Its Influence
on the Efficiency of the Thermally Activated Delayed Fluorescence
of Dopant Molecules. J. Phys. Chem. C.

[53] Kachwal V., Tan J. C. (2023). Stimuli-Responsive
Electrospun Fluorescent Fibers Augmented
with Aggregation-Induced Emission (AIE) for Smart Applications. Advanced Science.

[54] Yang J., Fang M., Li Z. (2020). Organic Luminescent Materials: The
Concentration on Aggregates from Aggregation-Induced Emission. Aggregate.

[55] Wang Z., Yang T., Dong S., Wen Z., Xu H., Miao Y., Wang H., Yu J. (2022). Anthracene
and Carbazole
Based Asymmetric Fluorescent Materials for High-Efficiency Deep-Blue
Non-Doped Organic Light Emitting Devices with CIEy = 0.06. Dyes Pigm..

[56] Hsieh K., Zhuang Y., Huang J., Wei Z., Zhang Y., Lee J., Chiu T., Leung M. (2024). Enhancing
Triplet–Triplet
Annihilation Upconversion Performance Through Anthracene–Carbazole
Interactions for Organic Optoelectronic Applications. Adv. Photonics Res..

[57] Qiu X., Tian G., Lin C., Pan Y., Ye X., Wang B., Ma D., Hu D., Luo Y., Ma Y. (2021). Narrowband Emission from Organic Fluorescent Emitters
with Dominant
Low-Frequency Vibronic Coupling. Adv. Opt. Mater..

[58] Bialas A. L., Spano F. C. (2022). A Holstein-Peierls Approach to Excimer
Spectra: The
Evolution from Vibronically Structured to Unstructured Emission. J. Phys. Chem. C.

[59] Wegmann G., Schweitzer B., Hopmeier M., Oestreich M., Giessen H., Mahrt R. F. (1999). Conjugated Polymer Lasers: Emission
Characteristics and Gain Mechanism. Phys. Chem.
Chem. Phys..

[60] Kim Y., Bouffard J., Kooi S. E., Swager T. M. (2005). Highly Emissive
Conjugated Polymer Excimers. J. Am. Chem. Soc..

[61] Thadathilanickal T. D., Paul M., Karunakaran V. (2023). Ultrafast Intermolecular Energy Transfer
in OLED Materials: Excited-State Dynamics of a Blend of Poly­(Vinylcarbazole)
and Oxadiazole Derivative in Solution and Film States. J. Phys. Chem. C.

[62] De
Sainte Claire P. (2006). Molecular Simulation of Excimer Fluorescence in Polystyrene
and Poly­(Vinylcarbazole). J. Phys. Chem. B.

[63] Musser A. J., Rajendran S. K., Georgiou K., Gai L., Grant R. T., Shen Z., Cavazzini M., Ruseckas A., Turnbull G. A., Samuel I. D. W., Clark J., Lidzey D. G. (2017). Intermolecular States
in Organic Dye Dispersions: Excimers vs. Aggregates. J. Mater. Chem. C.

[64] Osaheni J. A., Jenekhe S. A. (1995). Electroactive and Photoactive Rod-Coil Copolymers:
Design, Synthesis, and Supramolecular Regulation of Photophysical
Properties. J. Am. Chem. Soc..

[65] Kulkarni A. P., Kong X., Jenekhe S. A. (2004). Fluorenone-Containing
Polyfluorenes
and Oligofluorenes: Photophysics, Origin of the Green Emission and
Efficient Green Electroluminescence. J. Phys.
Chem. B.

[66] Liu Y., Tao X., Wang F., Shi J., Sun J., Yu W., Ren Y., Zou D., Jiang M. (2007). Intermolecular Hydrogen Bonds Induce
Highly Emissive Excimers: Enhancement of Solid-State Luminescence. J. Phys. Chem. C.

[67] Bhowmik S., Mondal D., Arora K., Neelakandan P. P., Sen P. (2025). Effect of Halogenation on the Photophysics of Salicylideneimine-Boron
Compound: An Unusual Behaviour with Bromination. J. Photochem. Photobiol., A.

[68] Demirbay B., Baryshnikov G., Haraldsson M., Piguet J., Ågren H., Widengren J. (2023). Photo-Physical Characterization of High Triplet Yield
Brominated Fluoresceins by Transient State (TRAST) Spectroscopy. Methods Appl. Fluoresc..

[69] Doi M., Liu H., Ando S. (2025). Prolonged Irradiation-Induced Delayed Luminescence
of PMMA-Dispersed Imide Compounds Containing Ether- and Thioether-Bridged
Cores. Mater. Chem. Front..

[70] Berezin M.
Y., Achilefu S. (2010). Fluorescence
Lifetime Measurements and Biological Imaging. Chem. Rev..

