# Supplementary material for: Enhancing Blue Emission in Poly(N‑vinylcarbazole): Synthesis, Functionalization with Anthracene, and Mitigation of Aggregation-Caused Quenching
Source: ACS Omega. 2025 Aug 21;10(34):39250–62. doi: 10.1021/acsomega.5c06357 (PMC12409685; doi:10.1021/acsomega.5c06357)
Supplement: Supplementary file 1 [file ao5c06357_si_001.pdf]

Support Information

For

Enhancing Blue Emission in Poly(N-vinylcarbazole): Synthesis, Functionalization with Anthracene, and Mitigation of Aggregation-Caused Quenching

Daniela Corrêa Santos, Gabriel de Sousa Barros, Maria de Fátima Vieira Marques*

Instituto de Macromoléculas Professora Eloisa Mano, Universidade Federal do Rio de Janeiro (IMA/UFRJ), Rio de Janeiro, RJ, Brazil

*Email: fmarques@ima.ufrj.br

1 NMR Analysis

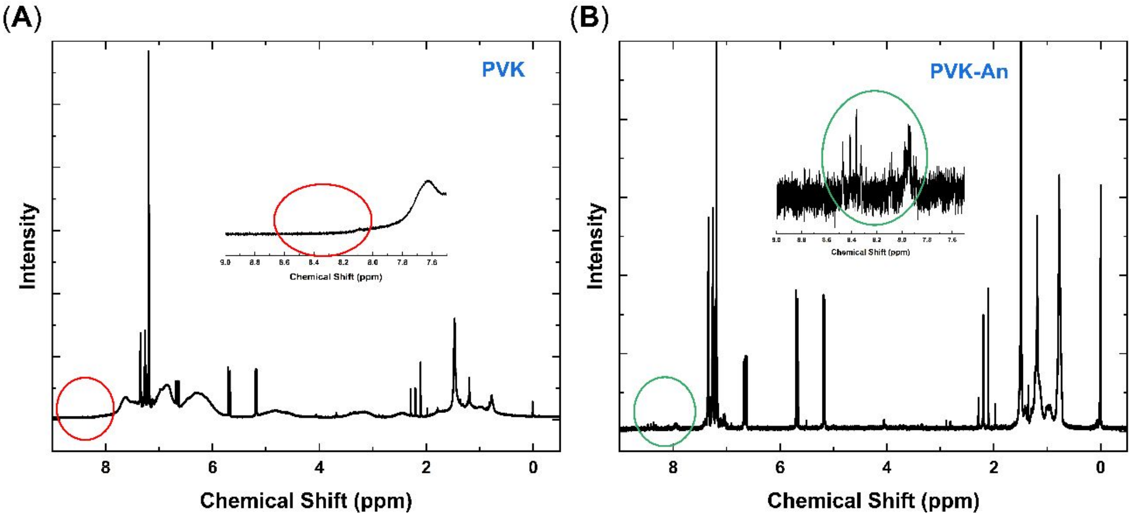

Figure S1. Zoomed-in ^{1}H-NMR Spectrum of (A) PVK and (B) PVK-An (9–7.5 ppm Region).

2 Photophysical Investigation

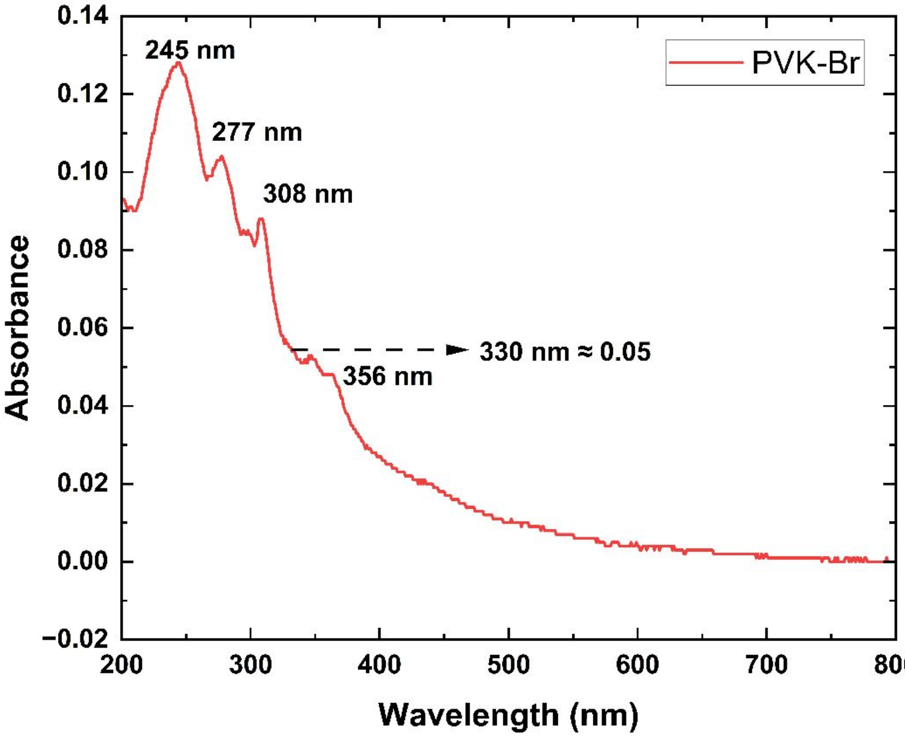

Figure S2. UV-vis spectrum of PVK-Br recorded on thin film.

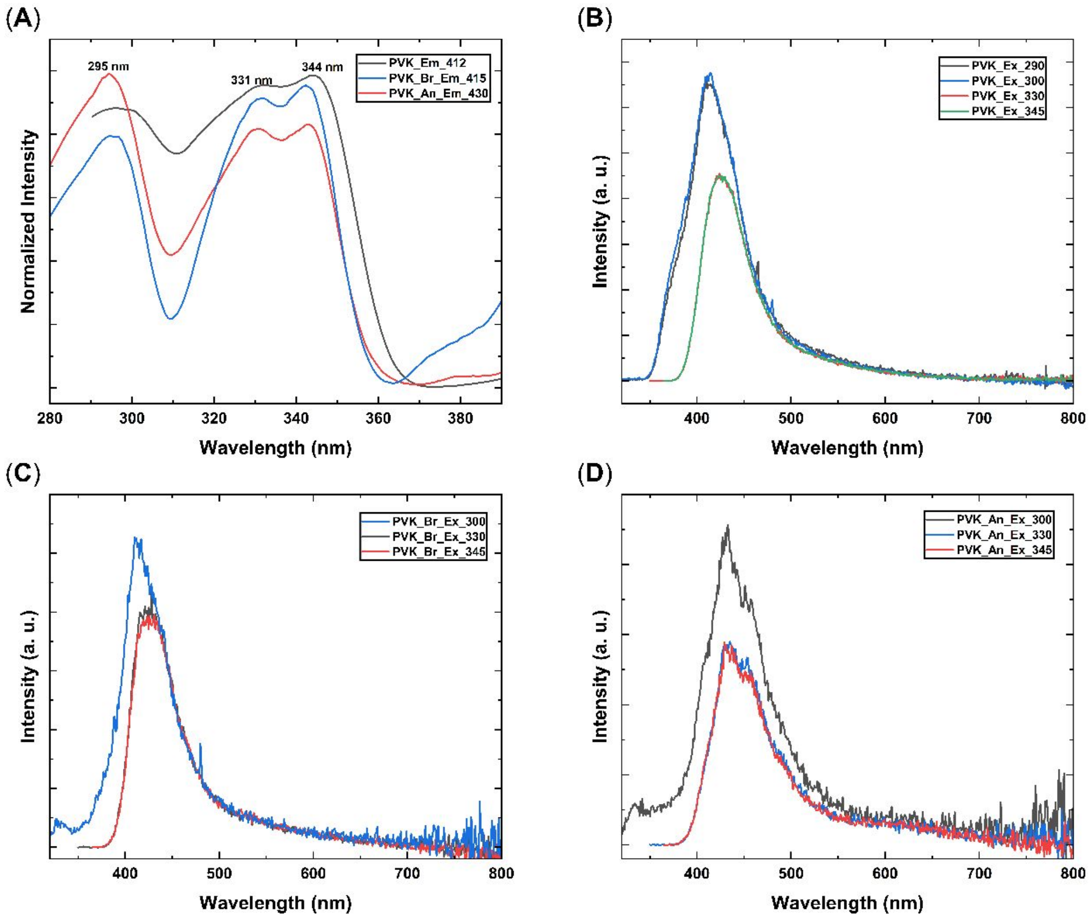

Figure S3. (A) Excitation spectra of the neat polymers in thin-film at λ_{em} = 412 nm for PVK, 415 nm for PVK-Br and 430 nm for PVK-An; (B) PVK emission spectra at λ_{ex} = 290, 300, 330 and 345 nm; (C) PVK-Br emission spectra at λ_{ex} = 300, 330, 345 nm and (D) PVK-An emission spectra at λ_{ex} = 300, 330 and 345 nm.

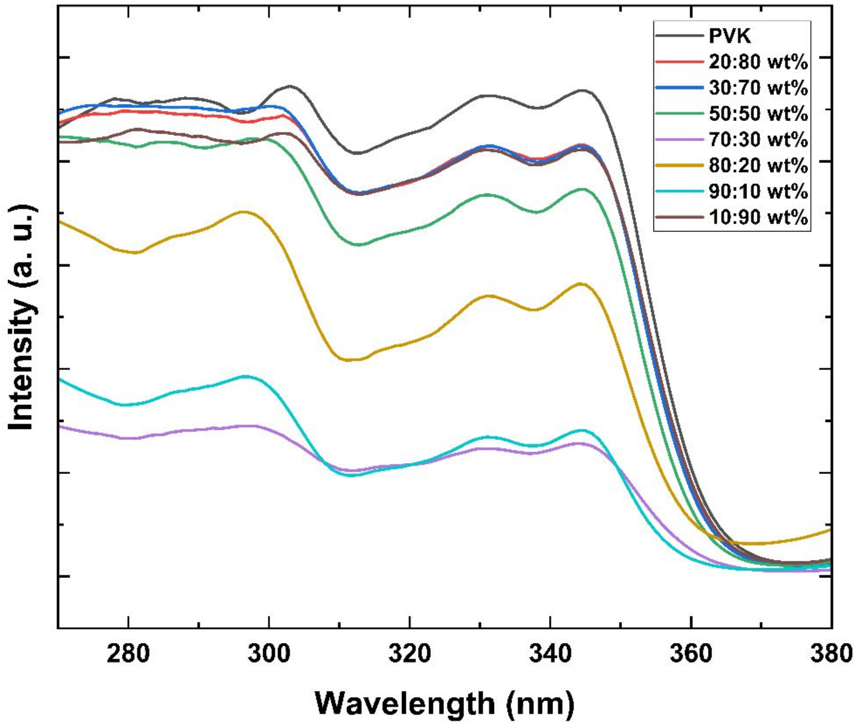

Figure S4. Excitation spectra of the PMMA:PVK matrix recorded in thin-film at different in different ratios and emission wavelength at 406 nm.

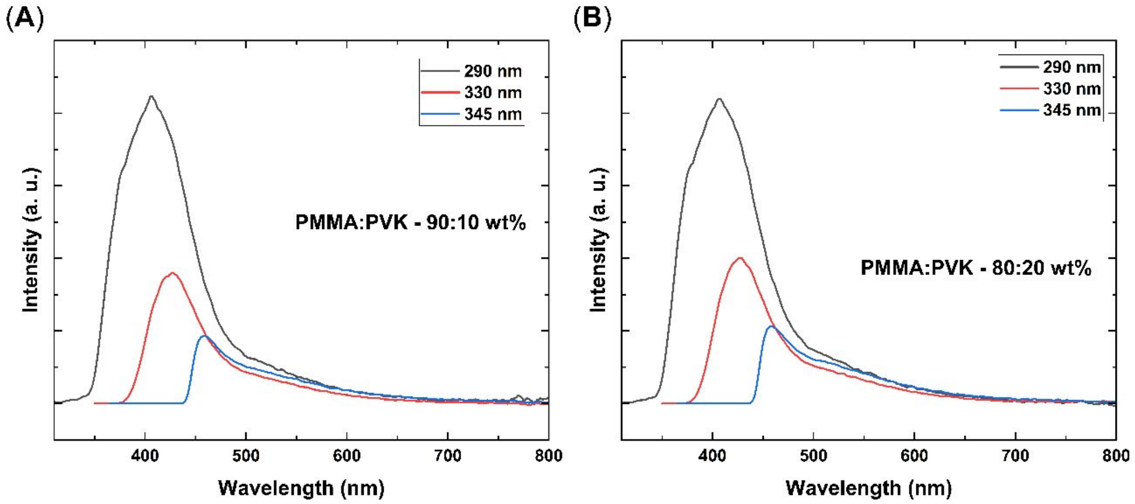

Figure S5. Emission spectra of the PMMA:PVK matrix recorded in thin-film at different excitations wavelengths. (A) PMMA:PVK 90:10 wt% and (B) PMMA:PVK 80:20 wt%.

3 Time-Resolved Photoluminescence

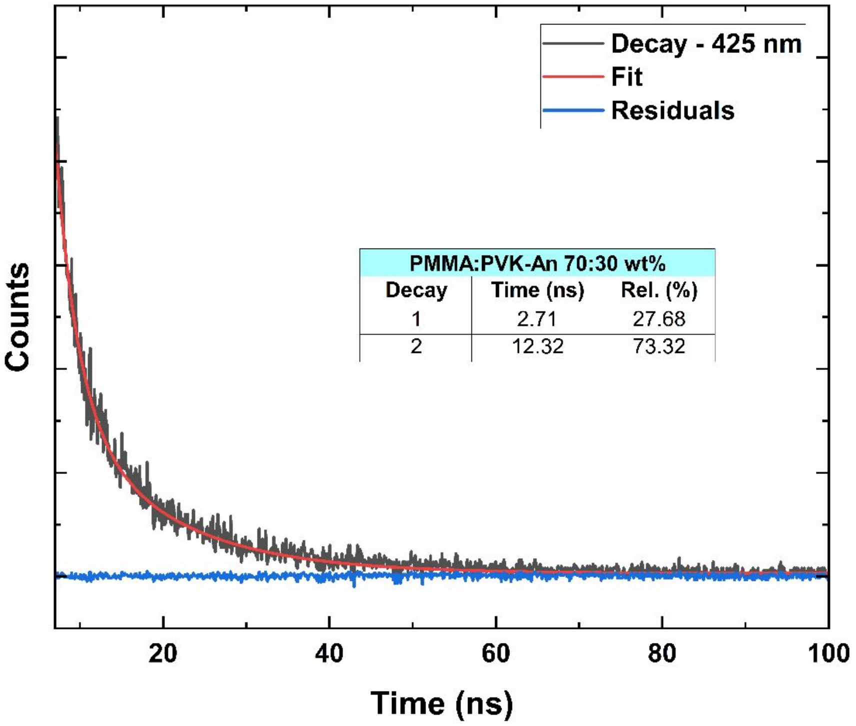

Figure S6. Time-Resolved photoluminescence spectrum of the PMMA:PVK-An 70:30 wt% matrix, recorded on thin film at the emission wavelength of 425 nm.

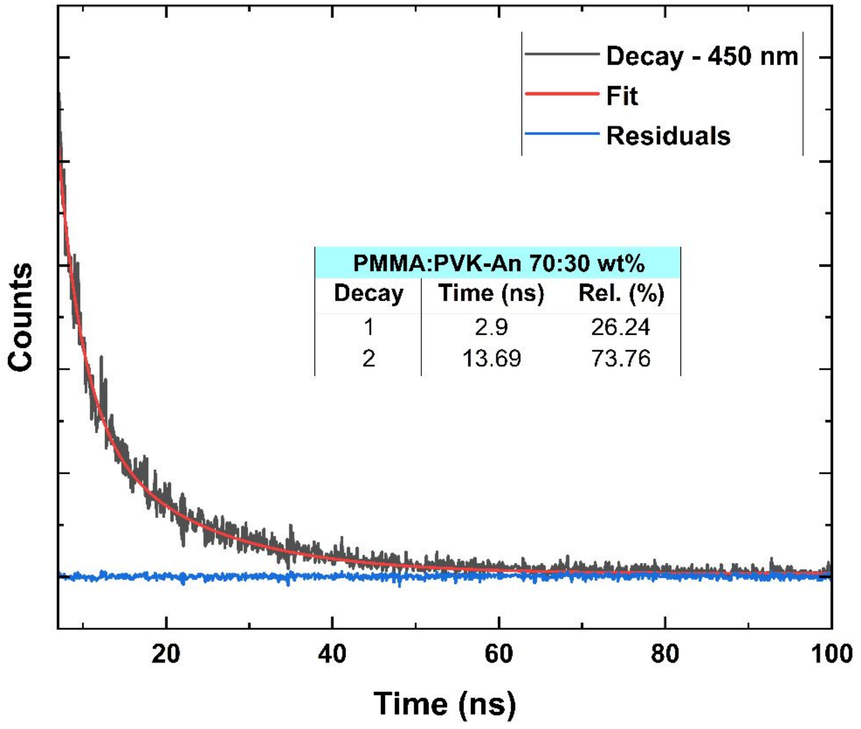

Figure S7. Time-Resolved photoluminescence spectrum of the PMMA:PVK-An 70:30 wt% matrix, recorded on thin film at the emission wavelength of 450 nm.

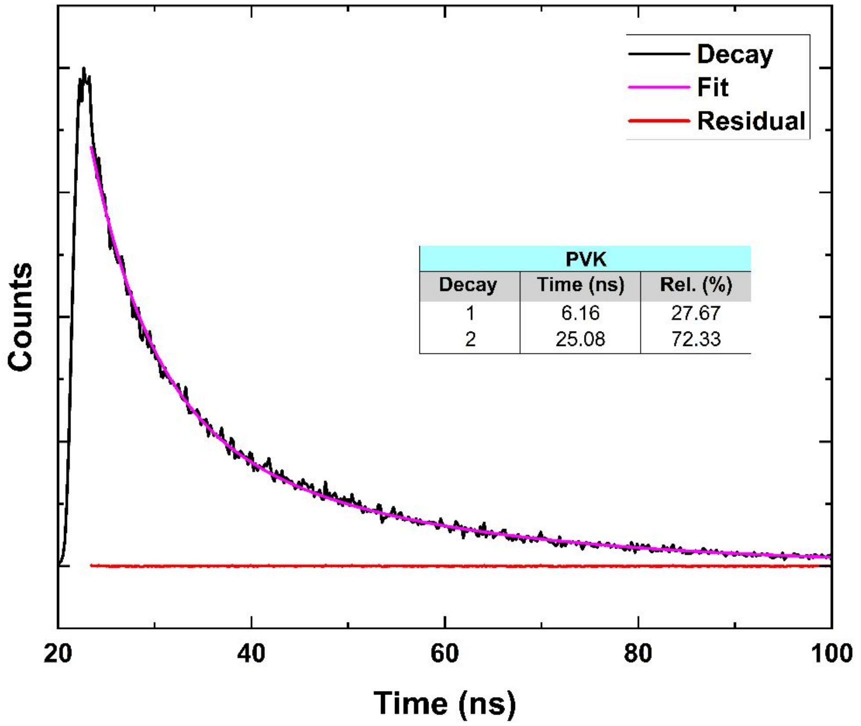

Figure S8. Time-Resolved photoluminescence spectrum of PVK, recorded on thin film.

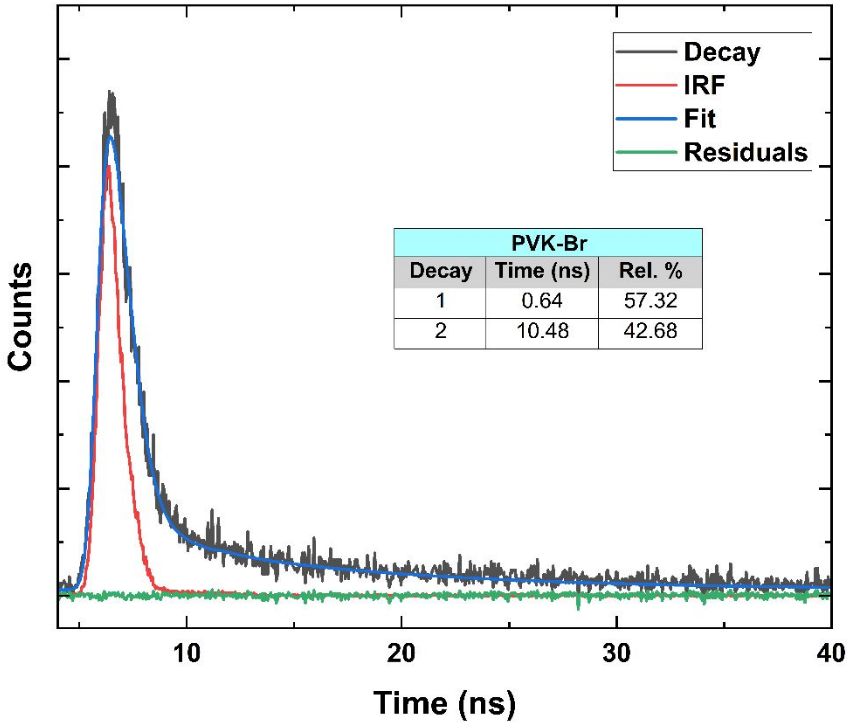

Figure S9. Time-Resolved photoluminescence spectrum of PVK-Br, recorded on thin film.

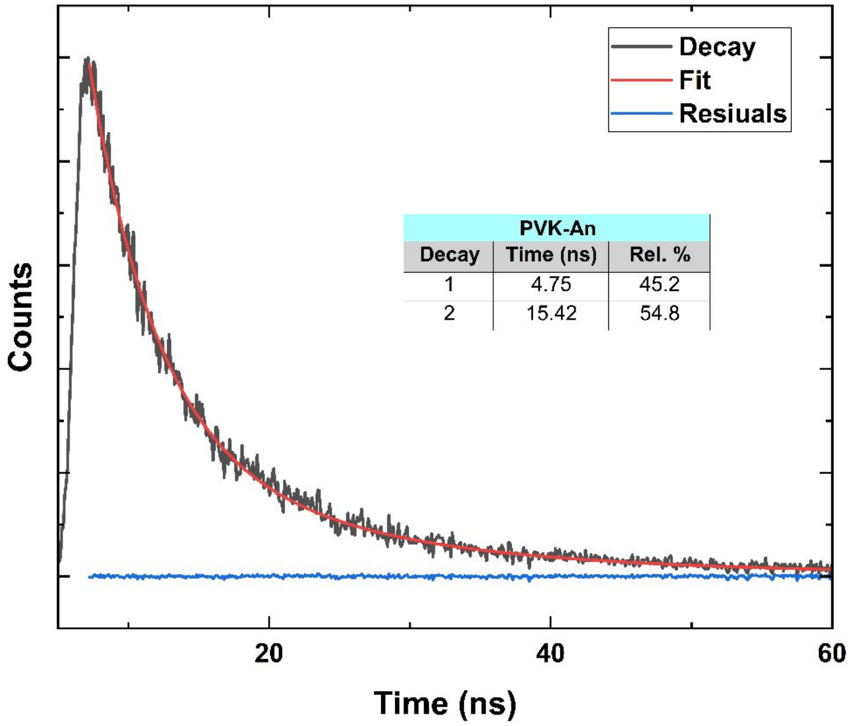

Figure S10. Time-Resolved photoluminescence spectrum of PVK-An, recorded on thin film.

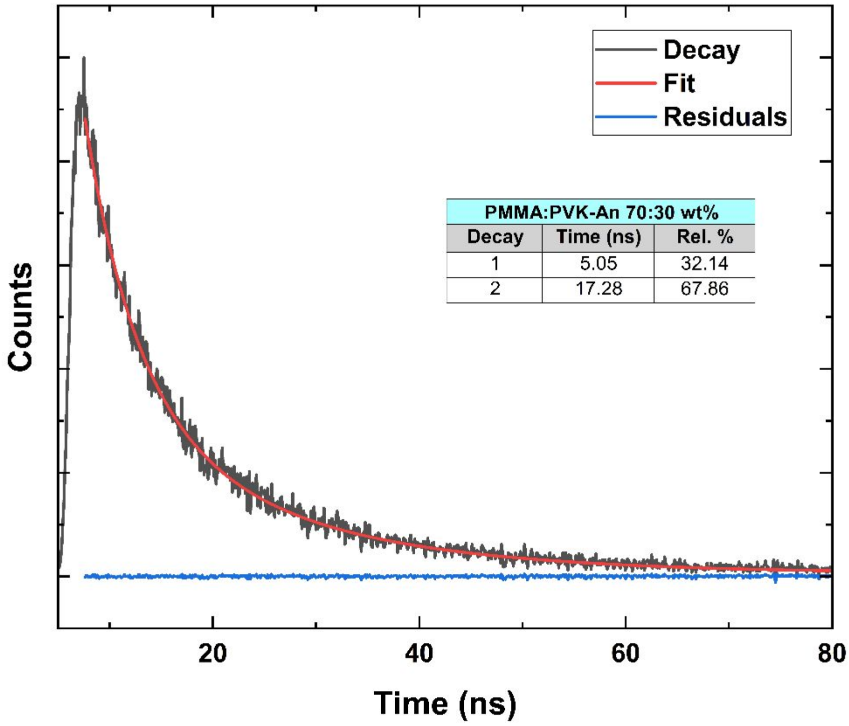

Figure S11. Time-Resolved photoluminescence spectrum of PMMA:PVK-An 70:30 wt%, recorded on thin film at wavelength of 425 nm.
